# Determinants of initial inhaled corticosteroid use in patients with GOLD A/B COPD: a retrospective study of UK general practice

**DOI:** 10.1038/s41533-017-0040-z

**Published:** 2017-06-29

**Authors:** James D. Chalmers, Abigail Tebboth, Alicia Gayle, Andrew Ternouth, Nick Ramscar

**Affiliations:** 1 Scottish Centre for Respiratory Research, Ninewells Hospital and Medical School, University of Dundee, Dundee, Scotland; 2 Boehringer Ingelheim Ltd., Bracknell, Berkshire UK

## Abstract

Initial use of inhaled corticosteroid therapy is common in patients with Global Initiative for Chronic Obstructive Lung Disease (GOLD) A or B chronic obstructive pulmonary disease, contrary to GOLD guidelines. We investigated UK prescribing of inhaled corticosteroid therapy in these patients, to identify predictors of inhaled corticosteroid use in newly diagnosed chronic obstructive pulmonary disease patients. A cohort of newly diagnosed GOLD A/B chronic obstructive pulmonary disease patients was identified from the UK Clinical Practice Research Datalink (June 2005–June 2015). Patients were classified by prescribed treatment, with those receiving inhaled corticosteroid-containing therapy compared with those receiving long-acting bronchodilators without inhaled corticosteroid. In all, 29,815 patients with spirometry-confirmed chronic obstructive pulmonary disease were identified. Of those prescribed maintenance therapy within 3 months of diagnosis, 63% were prescribed inhaled corticosteroid-containing therapy vs. 37% prescribed non-inhaled corticosteroid therapy. FEV_1_% predicted, concurrent asthma diagnosis, region, and moderate exacerbation were the strongest predictors of inhaled corticosteroid use in the overall cohort. When concurrent asthma patients were excluded, all other co-variates remained significant predictors. Other significant predictors included general practitioner practice, younger age, and co-prescription with short-acting bronchodilators. Trends over time showed that initial inhaled corticosteroid prescriptions reduced throughout the study, but still accounted for 47% of initial prescriptions in 2015. These results suggest that inhaled corticosteroid prescribing in GOLD A/B patients is common, with significant regional variation that is independent of FEV_1_% predicted.

## Early-stage chronic lung disease: overuse of inhaled steroids in the UK

Inhaled steroids are often prescribed to early-stage chronic lung disease patients in the UK despite guidelines to the contrary. Patients newly diagnosed with early-stage chronic obstructive pulmonary disease (COPD) should not be prescribed inhaled corticosteroids (ICS), because they carry an increased risk of side effects such as pneumonia and osteoporosis. ICS should be reserved for patients with severe COPD and frequent exacerbations. James Chalmers at the Scottish Centre for Respiratory Research, Dundee, and co-workers examined prescribed medication data from the UK spanning 10 years, to determine key predictors of ICS prescription during early-stage COPD. Of 29,815 patients identified, an average of 63% were prescribed ICS upon diagnosis, regardless of disease severity. Younger patients were more likely to receive ICS, possibly due to co-morbidity with chronic asthma, and particular UK regions and medical practices prescribed ICS more readily than others.

## Introduction

Current international guidelines for chronic obstructive pulmonary disease (COPD) recommend long-acting inhaled bronchodilators, including β2-agonists (LABA) and anti-muscarinic agents (LAMA), as maintenance therapies.^1^ These agents can be given as monotherapy, as combination bronchodilators, or in combination with inhaled corticosteroids (ICS) for the symptomatic management of COPD. The Global Initiative for Chronic Obstructive Lung Disease (GOLD) 2016 guidelines recommend that ICS therapy is reserved for COPD patients with severe/very severe disease and/or frequent or severe exacerbations.^1^ They do not recommend ICS therapy for GOLD stage A or B COPD patients. Similarly, NICE clinical guidelines for COPD (CG101) recommend ICS in combination with LABA if FEV_1_ < 50% predicted, and ICS in combination with LAMA + LABA for patients who remain breathless or have exacerbations despite taking LABA + ICS.^2^ However, a significant dissociation has been reported between guideline recommendations and clinical practice,^3, 4^ with common use of ICS in patients with GOLD stage A and B COPD.^5^ Further research also suggests that many patients are prescribed ICS therapy at their initial COPD diagnosis, regardless of disease severity.^6^ Recent randomised controlled trials show combined bronchodilator treatment is superior to ICS/LABA in lung function improvement, symptomatic benefit, and reduction in exacerbations, including in patients with GOLD stage B disease.^7–9^ ICS therapy is important in the treatment of asthma-COPD overlap (ACO), and ICS combinations should be the default treatment for patients with features of both asthma and COPD.^1, 10^ They are not appropriate, however, for patients with GOLD A and B COPD based on the available treatment guidelines and clinical evidence.

Inappropriate ICS treatment carries important risks, with randomised controlled trials demonstrating an increased incidence of pneumonia, fractures, and other side effects compared to long-acting bronchodilators.^11–15^ Reducing inappropriate ICS prescribing in early-stage COPD is therefore a key quality improvement objective, but one that has not been achieved despite a consistent message from national and international guidelines. It is therefore essential to understand what drives initial use of ICS in early COPD; as such the aim of this study was to identify factors that were independently associated with UK prescribers’ decisions to prescribe ICS therapies in patients with early COPD.

## Results

A total of 29,815 patients with spirometry-confirmed COPD of GOLD stage A or B were identified during the study period (see Supplementary Table S1 and Supplementary Fig. S1 in the Supplementary Information for baseline characteristics and patients included in the study). There were slightly more male than female patients (54 vs. 46%), and the most common age category was 60–69 years (mean age 67 years). The most common comorbidities were concurrent asthma (20%), diabetes (9%), myocardial infarction (7%), and osteoporosis/osteopenia (7%). Eosinophilia was recorded as present in 23% of patients. The majority of patients were current or ex-smokers (83%) and 1% of patients had one moderate exacerbation in the year prior to diagnosis.

Fewer patients were observable in the cohort in the latter years of the study (2009–2015); this is most likely due to variation in the frequency of data uploading by practices to the Clinical Practice Research Datalink (CPRD). The region with the largest volume of data was North West England, although all UK regions were represented in the study.

**Fig. 1:**
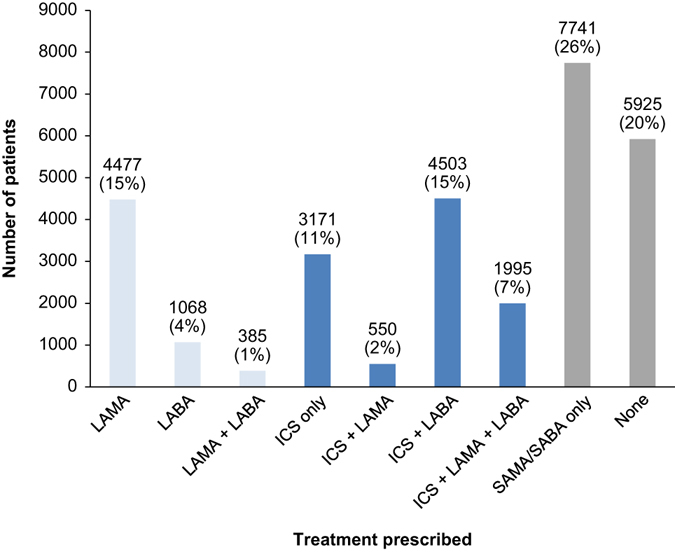
Number of patients by therapy combination at COPD diagnosis (overall cohort). *COPD* chronic obstructive pulmonary disease, *ICS* inhaled corticosteroids, *LABA* long-acting beta-agonists, *LAMA* long-acting muscarinic antagonists, *SABA* short-acting beta-agonists, *SAMA* short-acting muscarinic antagonists

The therapies prescribed to patients in the overall cohort within 3 months of COPD diagnosis are displayed in Fig. 1. The number of patients initially prescribed ICS-containing therapy is considerably higher than the number prescribed long-acting, non-ICS therapy (34 vs. 20%, respectively).

**Fig. 2:**
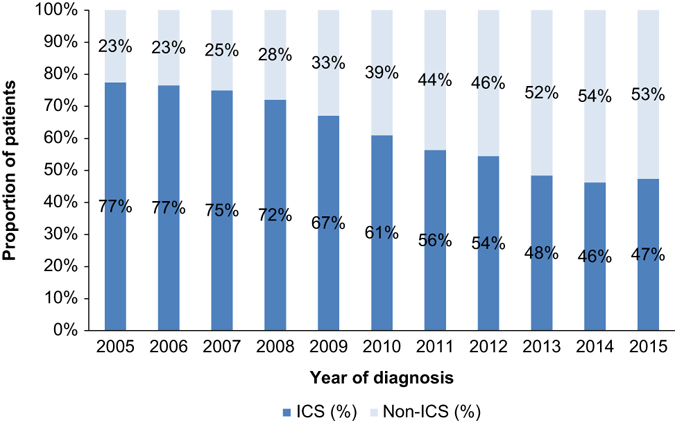
Prescribing of ICS vs. non-ICS-containing therapies over time (as a percentage of maintenance treatment). *ICS* inhaled corticosteroids

Of the patients prescribed maintenance therapy (i.e., excluding patients prescribed short-acting therapy or receiving no treatment), an average 63% were prescribed ICS-containing therapy vs. 37% prescribed non-ICS therapy. There was a strong trend to reduction of initial ICS prescribing over time, falling from 77% in 2005 to 47% at the end of the study (*p* < 0.0001). The greatest proportion of patients was prescribed ICS-containing maintenance therapy if initiating treatment prior to 2009 (Fig. 2).

Baseline characteristics in the ICS group were compared with the non-ICS group (Table 1). Patients in the ICS group were slightly more likely to be female than the non-ICS group (48 vs. 46% female; *p* = 0.043), were likely to be younger (*p* < 0.0001), and more likely to be diagnosed in the earlier years of the study (*p* < 0.0001). Patients in the ICS group were also more likely to be diagnosed with concurrent asthma (35 vs. 12%; *p* < 0.0001) and have moderate exacerbations in the year prior to diagnosis (2 vs. 1%; *p* = 0.0004) and less likely to be current or ex-smokers (81 vs. 86%; *p* < 0.0001) or have a history of myocardial infarction (6.4 vs. 7.5%; *p* = 0.006).

**Table 1:** Baseline characteristics of patients in the ICS and non-ICS groups

Characteristic	ICS group		Non-ICS group		*p*-value
	*N*	%	*N*	%	
All patients	10,219	63.3	5930	36.7	
Gender (% male)	5346	52.3	3200	54.0	**0.043** ^a^
Age					
40–49 years	778	7.6	329	5.5	<**0.0001** ^b^
50–59 years	1977	19.3	1063	17.9	
60–69 years	3389	33.2	1979	33.4	
70–79 years	2848	27.9	1819	30.7	
80–89 years	1149	11.2	708	11.9	
>90 years	78	0.8	32	0.5	
Year of diagnosis					
2005	601	77.4	175	22.6	<**0.0001** ^b^
2006	1332	76.6	408	23.4	
2007	1309	75.0	437	25.0	
2008	1300	72.1	503	27.9	
2009	1241	67.0	610	33.0	
2010	1105	61.0	707	39.0	
2011	1051	56.3	815	43.7	
2012	922	54.5	771	45.5	
2013	704	48.4	750	51.6	
2014	527	46.2	613	53.8	
2015	127	47.4	141	52.6	
Comorbidities					
Asthma					
Historic	331	3.2	162	2.7	0.0709
Concurrent	3568	34.9	730	12.3	**<0.0001**
Stroke	311	3.0	195	3.3	0.389
MI	**651**	**6.4**	**445**	**7.5**	**0.006**
Diabetes	936	9.2	565	9.5	0.437
Osteoporosis/osteopenia	723	7.1	457	7.7	0.137
Eosinophilia	2514	24.6	1386	23.4	0.079
Pneumonia	360	3.5	193	3.3	0.366
Smoking (current or ex)^c^	8229	80.5	5112	86.2	**<0.0001**
BMI					
Missing	55	0.5	44	0.7	0.9995
Underweight	379	3.7	235	4.0	
Normal	3360	32.9	1936	32.6	
Overweight	3640	35.6	2081	35.1	
Obese	2785	27.3	1634	27.6	
FEV_1_% predicted					
80–100% (GOLD 1)	3433	33.6	1871	31.6	**0.0077**
50–80% (GOLD 2)	6786	66.4	4059	68.4	
Moderate exacerbations^d^	157	1.5	52	0.9	**0.0004**
Region					
North East	194	1.9	173	2.9	**<0.0001**
North West	1525	14.9	865	14.6	
Yorkshire and The Humber	284	2.8	118	2.0	
East Midlands	273	2.7	76	1.3	
West Midlands	932	9.1	500	8.4	
East of England	700	6.8	329	5.5	
South West	901	8.8	409	6.9	
South Central	986	9.6	601	10.1	
London	980	9.6	483	8.1	
South East Coast	1005	9.8	508	8.6	
Northern Ireland	539	5.3	328	5.5	
Scotland	770	7.5	750	12.6	
Wales	1130	11.1	790	13.3	

*BMI* body mass index, *FEV*_*1*_ forced expiratory volume in 1 s, *GOLD* Global Initiative for Chronic Obstructive Lung Disease, *MI* myocardial infarction

Bold values indicate statistical significance

^a^ Chi-squared test

^b^ Cochran Armitage test for trend

^c^ Ex-smoker and current smoker were pooled due to limitations in CPRD for distinguishing between these two groups

^d^ In the year prior to COPD diagnosis (the index date)

**Fig. 3:**
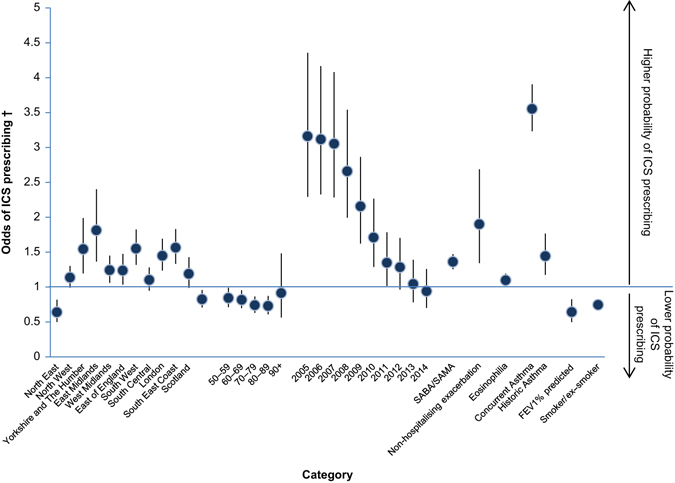
Logistic regression model—odds of prescribing ICS to GOLD A/B COPD patients at diagnosis (overall cohort). *SABA* short-acting beta-agonists, *SAMA* short-acting muscarinic antagonists. † Comparisons were made against a reference category in the case of region (Wales), age (40–49 years), and year of diagnosis (2015)

These variables were tested in a logistic regression analysis to identify the factors that affect prescribing of ICS-containing therapy. In the overall cohort, FEV_1_% predicted (*p* = 0.0002), moderate exacerbation in the year prior to diagnosis (*p* = 0.0004), UK region (*p* < 0.0001), age group (*p* = 0.0017), year of diagnosis (*p* < 0.0001), co-prescription of short-acting beta-agonists (SABA)/short-acting muscarinic antagonists (SAMA) therapy (*p* < 0.0001), concurrent asthma (*p* < 0.0001), historic asthma (*p* = 0.0006), eosinophilia (*p* = 0.0466), and current/ex-smoker (*p* < 0.0001) were all significantly associated with probability of ICS prescribing (Fig. 3). General Practitioner (GP) practice was also significantly associated with likelihood of ICS prescribing (*p* < 0.001); however, this could not be included in the model due to the small number of patients in many practices. Gender (*p* = 0.3635), stroke (*p* = 0.9763), myocardial infarction (*p* = 0.1043), diabetes (*p* = 0.8596), osteoporosis/osteopenia (*p* = 0.7969), history of pneumonia (*p* = 0.133), and body mass index (BMI) (*p* = 0.4792) were not significantly associated with ICS prescribing. After checking residuals, there were no violations of model assumptions.

A second analysis was also conducted, excluding patients with concurrent asthma. The proportion of patients in the ICS and non-ICS groups, as well as those prescribed SAMA/SABA therapy or no treatment, is shown in Supplementary Fig. S2 in the Supplementary Information. Of those prescribed maintenance therapy, 44% were prescribed non-ICS therapy and 56% were prescribed ICS-containing therapy.

**Fig. 4:**
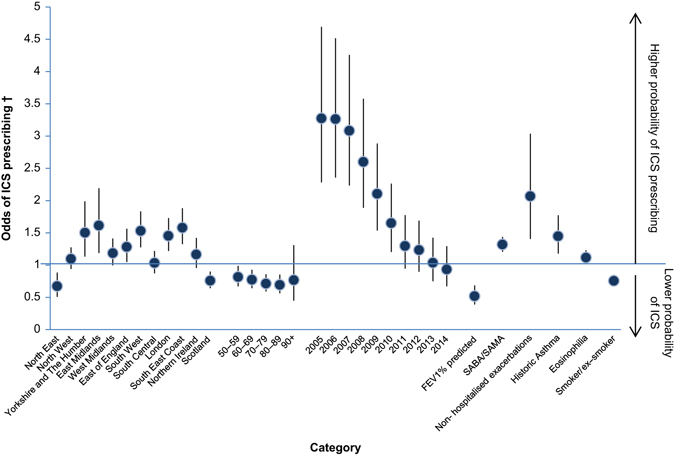
Logistic regression model excluding concurrent asthma patients. *SABA* short-acting beta-agonists, *SAMA* short-acting muscarinic antagonists. † Comparisons were made against a reference category in the case of region (Wales), age (40–49 years), and year of diagnosis (2015)

Logistic regression analysis in the cohort excluding patients with concurrent asthma showed that UK region (*p* < 0.0001), age group (*p* = 0.0045), year of diagnosis (*p* < 0.0001), FEV_1_% predicted (*p* < 0.0001), moderate exacerbation in the year prior to diagnosis (*p* = 0.0002), co-prescription of SABA/SAMA therapy (*p* < 0.0001), historic asthma (*p* = 0.0004), eosinophilia (*p* = 0.025), and current/ex-smoker (*p* < 0.0001) were still significantly associated with probability of ICS prescribing (Fig. 4).

A third analysis was conducted in the group of patients with possible ACO (i.e., COPD with asthma diagnosed in the previous 5 years). Logistic regression analysis in this cohort showed that UK region (*p* = 0.007), year of diagnosis (*p* < 0.0001), co-prescription of short-acting therapy (*p* < 0.0001), history of stroke (*p* = 0.0202), and current/ex-smoker (*p* = 0.0021) were significantly associated with probability of ICS prescribing (Supplementary Fig. S3).

### Sensitivity analysis

FEV_1_% predicted (as a continuous variable) and year of diagnosis were included as interaction terms in the model to determine if these varied significantly by region, and if they could be responsible for the differences in ICS prescribing by region. FEV_1_% predicted was found to be statistically insignificant (*p* = 0.1372), whereas year of diagnosis was significant (*p* < 0.001). The interaction between eosinophilia and concurrent and historic asthma was also investigated. Controlling for all variables, having baseline eosinophilia and concurrent (*p* = 0.6002) or historic asthma (*p* = 0.3849) was not associated with the odds of prescribing an ICS-containing therapy.

A third sensitivity analysis was conducted comparing predictors of ICS prescribing in diagnoses before and after 2009. This cut-off was chosen to investigate the impact of publication of the results of the TORCH study, which first identified the link between ICS use and pneumonia, leaving reasonable time to allow for implementation and recording in CPRD.^11^ Region, co-prescription with SABA/SAMA, concurrent and historic asthma, and smoker/ex-smoker were significant predictors both before and after 2009. FEV_1_% predicted, moderate exacerbations, and year of diagnosis became significant after 2009, whereas age group and myocardial infarction were only significant pre-2009.

## Discussion

### Main findings

This study suggests that a large number of patients with GOLD stage A or B COPD are prescribed ICS-containing therapy within 3 months of diagnosis. Over the duration of the study, around twice the number of patients were prescribed ICS-containing therapy rather than long-acting bronchodilators. When patients with concurrent asthma were excluded, the number of patients in the ICS group was still greater than the number in the non-ICS group (56 vs. 44% of those prescribed maintenance therapy). This suggests the need for major changes in UK prescribing practices in order to reduce inappropriate ICS use. The 2017 GOLD strategy has recently been published and reduces further the role of ICS by removing lung function from the criteria for GOLD C/D.^16^ Our analysis suggests that a major shift in prescribing will be required before UK practice is reflective of either the 2016 or 2017 GOLD strategy or the NICE guidelines.^1, 2, 16^ Trends over time show that prescribing practice is becoming more aligned with GOLD 2016 guidelines; ICS maintenance therapy prescriptions reduced from 77% in 2005 to 47% in 2015. This could reflect changes over the period of the study, including increased knowledge of side effects related to ICS use, such as pneumonia, fractures, osteoporosis, cataracts, tuberculosis, early onset diabetes, and bruising.^11, 14, 15, 17, 18^ It may also reflect the more recent availability of LAMA/LABA fixed-dose combinations, together with supporting clinical data that show they improve FEV_1_ and breathlessness symptoms and reduce exacerbations compared to ICS/LABA therapy in populations including GOLD B patients.^7–9, 19^ ICS have a role to play in patients who continue to experience exacerbations after treatment with bronchodilators.^1^ However, the overall results show that there is still a striking excess use of ICS in patients with GOLD A/B COPD, which persists in spite of accumulating evidence that there are safer and more efficacious alternatives in bronchodilator treatment. This disconnect between evidence and practice necessitates a clear understanding of what drives ICS prescribing practices.

Logistic regression analysis highlighted some factors that may be driving therapy decisions in these patients. In the overall cohort, FEV_1_% predicted, moderate exacerbation, UK region, age group, year of diagnosis, co-prescription of short-acting therapy, concurrent asthma, historic asthma, eosinophilia, and smoking status were significantly associated with ICS prescribing. When patients with concurrent asthma were excluded from the analysis, all other variables remained significant predictors. These predictors have also changed over the study duration, with FEV_1_% predicted, moderate exacerbations, and year of diagnosis becoming significant after 2009, suggesting prescribing drivers are changing with time. It is also interesting to note that osteoporosis, diabetes, and pneumonia did not drive reduced ICS use, despite being known side effects of long-term ICS use.^11, 14, 15, 17, 18^

These results provide some insight and allow some speculation on how ICS therapies are currently being used. FEV_1_ and history of exacerbations are markers of the severity and activity of COPD. Our results suggest that patients with more severe COPD and patients with a history of one exacerbation are more likely to receive ICS. This may reflect a lack of knowledge of the cut-offs used in GOLD guidelines to determine ICS use (FEV_1_ < 50% predicted and >1 exacerbation) or a lack of confidence in bronchodilators to prevent exacerbations despite available evidence to the contrary.^7–9, 19^ The strong association with asthma suggests one of two possibilities: that the presence of ACO is very common and ICS is being used appropriately in those patients where both conditions are present, or that diagnostic confusion between these two conditions is leading to overuse of ICS as physicians look to cover both possibilities. Confusion would be understandable in primary care, as there is no gold standard test to distinguish between asthma and COPD, and diagnosis is reliant on spirometry.

**Fig. 5:**
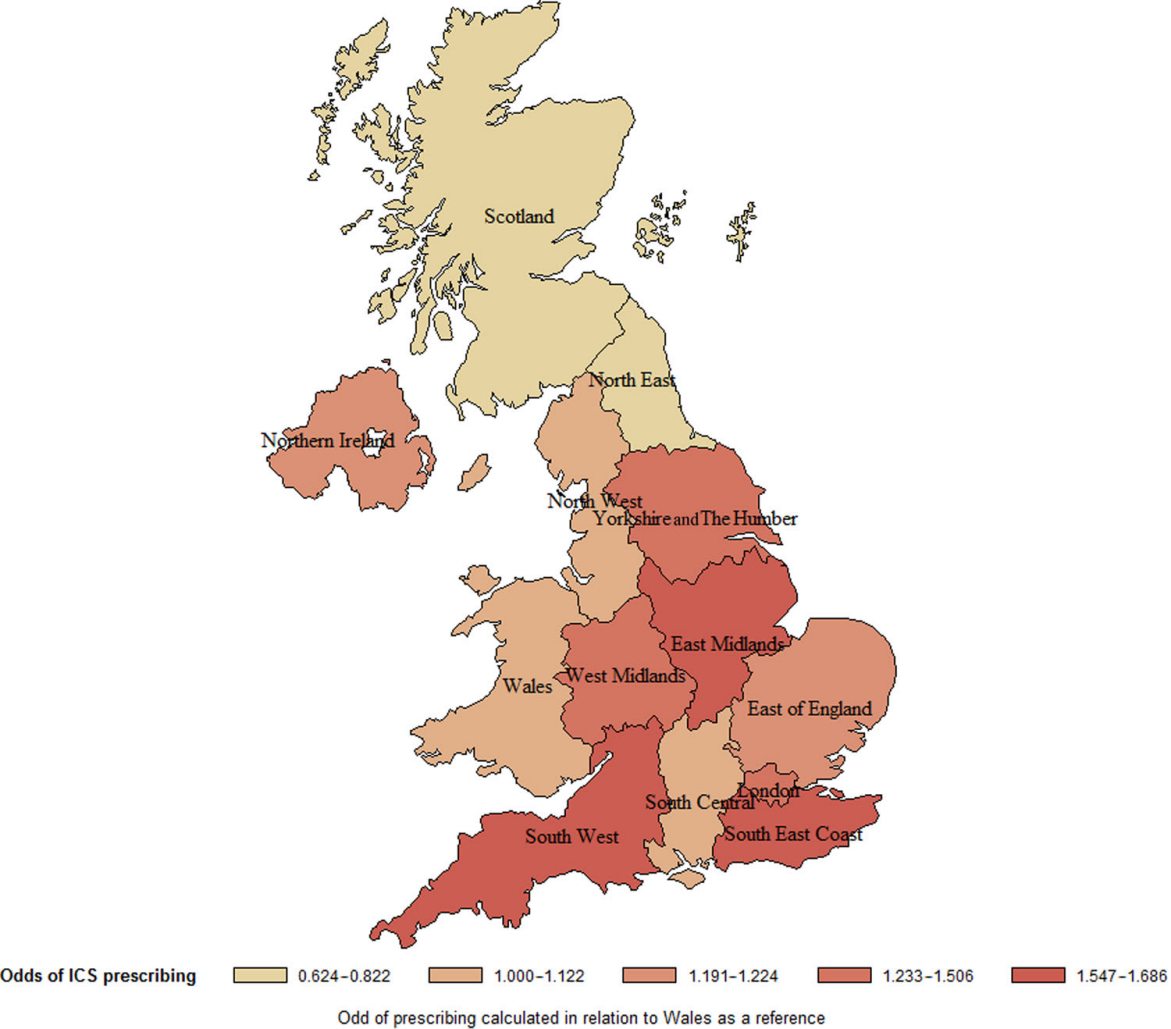
Odds of ICS prescription at COPD diagnosis by UK region. Created by the authors using SAS software version 9.4

UK region was also a significant predictor of ICS prescribing in patients with GOLD A or B COPD and we also identified clear patterns within regions suggesting that particular GP practices were high ICS users compared to others. This suggests a strong influence of personal prescribing preferences, local guidelines, and local culture in determining ICS use.^20^ In North East England and Scotland, patients are less likely to receive an initial prescription for ICS combinations than the comparator region (Wales). In the remaining regions, patients are more likely to receive ICS-containing therapy, with up to 1.7 times increased probability of prescription in three regions (East Midlands, South West, and South East Coast; see Fig. 5). This variation could not be explained by differences in disease severity, as FEV_1_% predicted was not significantly different between regions (*p* = 0.1372). Some centres contributed more data in the earlier or later years of the study and so some of the regional data may be biased by changes in prescribing trends over time. Yorkshire and The Humber and East Midlands, for example, were skewed towards the earlier years of the study when ICS prescribing was more frequent (Supplementary Fig. S4). However, it does not explain all the variations as Scotland shows very similar distribution to Wales, and the North East had low rates of ICS prescribing despite having the majority of its data from the earlier years of the study. Variation in respiratory care between UK regions is being monitored by the NHS as part of the Right Care and Atlas of Variation initiatives. The latest report showed considerable variation in a number of metrics related to respiratory diseases, including a seven-fold difference in the rate of COPD emergency admissions to hospital.^21^ This is consistent with our finding of regional variation in prescribing behaviour, and highlights the importance placed on unwarranted variation by the NHS.

Other significant predictors show that younger patients, with lower likelihood of being current or ex-smokers, were more likely to be prescribed ICS-containing therapy. This is highly suggestive of a diagnostic overlap with chronic asthma. Patients with historic asthma were also more likely to be treated with ICS. Improving diagnosis of asthma and clear guidance around the potential overlap between asthma and COPD could therefore lead to a reduction in the number of prescriptions of ICS therapy to patients diagnosed with mild or moderate COPD. Asthma diagnosis is a topic that is currently being considered by the National Institute for Health and Care Excellence, with an asthma diagnosis and monitoring guideline currently in development. This guideline could help GPs with a more systematic approach to the diagnosis of asthma and in turn with confidence in prescribing maintenance therapy to patients with mild or moderate COPD.

The finding that blood eosinophilia was associated with ICS use is intriguing. While this may be linked to asthma, eosinophilia remained a significant predictor of ICS use even after the exclusion of patients with a diagnosis of asthma. Although recent post hoc analyses of randomised controlled trials suggest that blood eosinophilia can identify a subgroup of patients that are responsive to ICS,^22–24^ there is no evidence that practitioners are currently using eosinophil counts to guide ICS use. This suggests that there are clinical characteristics associated with eosinophilia that lead physicians to prefer the use of ICS, independent of asthma diagnosis.

### Interpretation of findings in relation to previously published work

Our findings are consistent with previous studies on prescribing in COPD, which suggest that around 50% of patients with COPD are treated with ICS therapy.^4, 25^ A recent UK-based study by Gruffyd-Jones et al. also noted high prescribing rates of ICS therapies and major differences between actual prescribing and guideline recommendations.^4^ However, some differences exist, in terms of data source (CPRD vs. the Optimum Patient Database), time period (2005–2015 vs. 1997–2013), and main focus (initial ICS prescribing vs. initial prescription of any COPD therapy). These led to some differences in prescriptions recorded; e.g., we saw greater use of long-acting bronchodilators and an overall decrease in ICS-containing prescriptions over time, which was not observed in Gruffyd-Jones et al. We were also able to include variables that were not previously studied, including co-morbidities (osteoporosis/osteopenia, diabetes, cardiac disease, and eosinophilia) and demographic variables (UK region and GP practice). We were therefore able to identify that UK region and GP practice are both significant predictors of ICS prescription, and that these are independent of lung function.

This study considers initial prescriptions of ICS therapy to patients with mild/moderate COPD, which is one aspect of reducing inappropriate use of ICS. Another aspect of addressing this is the safe withdrawal of ICS therapy in appropriate patients, a topic that has been considered by other authors. Two recent randomised controlled trials, the largest being the WISDOM study and a real-life prospective study, have shown that ICS can be safely withdrawn in certain patients, and that this withdrawal may lead to reduction in ICS side effects such as risk of pneumonia.^19, 26–28^ Practical guides and algorithms have also been proposed to enable the withdrawal of ICS in appropriate patients, based on the currently available evidence.^29, 30^ The results of our study should be considered alongside the literature describing withdrawal of ICS, to provide a more complete picture of potential strategies for using maintenance therapy to maximise symptom relief and prevent harm in patients with COPD.

### Strengths and limitations of this study

Although CPRD is a well-recognised source for studies such as this, there are some limitations to the analysis. First, variables of interest are not always available in the database; we used medical research council dyspnoea score to calculate GOLD stage, as COPD CAT score was not available. Second, diagnoses are dependent on the physician entering the data and may not be standardised across the sample, although the impact of this is likely to be low as CPRD recording of COPD has been studied and found to be accurate.^31^ We are also not able to assess the effect of access or skill in spirometry on ICS prescribing, which will be variable across the UK, as having recorded spirometry data is required to allow GOLD A/B classification and hence inclusion in the study. Third, data are not available to the same extent and level of detail in all UK regions, as some areas have a higher COPD prevalence and some upload data on a more regular basis than others. However, as these differences occur on a random basis, this is less likely to lead to bias or affect the direction of the results. Finally, our study was based on GOLD classification, as this is well defined and relatively easy to study in CPRD. This could be a limitation, as not all GPs are familiar with the GOLD recommendations and some may have greater familiarity with the NICE guidelines, which are older and do not give as clear guidance for patient segmentation.^2^ However, as discussed above, neither NICE nor GOLD recommend use of ICS in patients with mild/moderate COPD.^1, 2, 16^ The study results should therefore be relevant to practices following NICE or GOLD recommendations.

### Implications for future research, policy, and practice

The results of this study highlight some aspects of the management of COPD in clinical practice, which may have implications for future policy. The regional variation in prescribing, for example, is a trend that may warrant examination and monitoring at national level, as this seems to be unrelated to clinical characteristics such as lung function. Unwarranted variation in the management of other primary care diseases has resulted in inclusion of new measures in the quality and outcomes framework, such as the inclusion of the eight care processes for diabetes.^32^ This may not be directly applicable for prescribing behaviours, given the number of factors influencing these decisions, but it does highlight the impact of national monitoring in influencing individual behaviour. Local initiatives, such as audits or reviews, could also help influence prescribing in order to improve adherence to guidelines and reduce the regional and local variation seen in our study.

As noted earlier, the GOLD 2017 strategy has recently been published, with a further reduced role for ICS.^16^ As a result, further research into what will happen in clinical practice following these new recommendations would also be interesting.

### Conclusions

A large proportion of patients with GOLD A or B COPD are given initial prescription of ICS therapy outside of guidelines in the UK. This varies according to differences in local prescribing practices (at practice and regional level), independently of lung function. The number of prescriptions outside of guidelines has decreased over recent years, possibly due to the availability of an alternative to ICS in terms of LABA/LAMA combinations and a greater awareness of adverse effects, but further work is still needed to ensure that the majority of patients are treated according to evidence-based guidelines.

## Methods

### Study design

We conducted a retrospective descriptive longitudinal study analysing data obtained from the UK CPRD. The CPRD provides a database of anonymised longitudinal clinical records from general practice, covering 689 GP practices in the UK, with 3.9 million people available for observation at the start of the study. The geographical distribution is representative of the UK population,^33^ and several studies have confirmed the high quality of the data and completeness of the clinical records.^31, 34–37^ As such it is recognised as a reliable source for investigating UK general practice and prescribing, and has been used in over 1500 publications to date (https://www.cprd.com/home/).

### Population

Patients of interest were those with a new diagnosis of GOLD A or B COPD registered at a CPRD practice during the study period (June 2005–June 2015). Patients also had to be aged 40 years or over at the date of diagnosis with at least 1 year of data prior to index entry. As the objective was to study the first prescription for maintenance therapy, we excluded patients already receiving maintenance treatment at study entry. Patients were also excluded if there was insufficient information in CPRD to confirm COPD diagnosis, or to calculate baseline GOLD stage. For further inclusion and exclusion criteria and definitions used, see Supplementary Table S2 in the Supplementary Material.

### Analysis

Patients were allocated to groups, based on the therapies they received within the first 3 months following COPD diagnosis. These groups included ICS-containing therapy (ICS only, ICS + LAMA, ICS + LABA, and ICS + LAMA + LABA), non-ICS-containing therapy (LAMA, LABA, and LAMA + LABA), short-acting therapy only (SABA or SAMA only), and none of the above (indicating no therapy of interest prescribed). Baseline data, including comorbidities and demographic information, were analysed descriptively. Variables of interest were summarised and compared between the ICS and non-ICS groups, including age, gender, smoking status, baseline BMI, year of COPD diagnosis, osteoporosis/osteopenia, diabetes, cardiac disease, historical or concurrent diagnosis of asthma, peripheral blood eosinophilia (defined using Read codes or a record of blood eosinophil count >0.4 × 10^9^/L), FEV_1_/FVC ratio, FEV_1_% predicted, UK region, GP practice, moderate exacerbation in the year prior to diagnosis, and history of pneumonia. These were identified by Read codes (see Supplementary Table S5 in the Supplementary Information). FEV_1_% predicted, FEV_1_/FVC ratio, and eosinophilia Read codes were confirmed using additional test information as described in Supplementary Table S6 (Supplementary Information). FEV_1_% predicted was capped at 100% for data quality purposes, as we cannot be sure of the accuracy of values above this point. Current and ex-smokers were pooled due to limitations in CPRD for distinguishing between these two groups. Statistical analysis was also carried out for baseline characteristics: gender, region, and comorbidities were compared using *χ*² tests; age group, year of diagnosis, and severity of airway obstruction were compared using Cochrane Armitage tests for trend (both with an *α* = 0.01).

Logistic regression analysis was used to investigate which factors affect prescribing of ICS-containing therapy. Variables were chosen for the model using a backwards stepwise method of selection with inclusion criteria of *p* < 0.05. Validity of the final model was assessed, and there were no violations of model assumptions after checking residuals. We also conducted sensitivity analysis to investigate interaction effects of suspected related variables, including: UK region and FEV_1_; UK region and year of diagnosis; and eosinophilia and concurrent or historic asthma.

Data were extracted using the online version of CPRD (CPRD-GOLD), and analysed using SAS software version 9.4. Missing data that occurred in covariates or descriptive variables were classified as their own level. Variables with >75% missing data were excluded from the analysis, with the exception of baseline characteristics.

### Data availability

The data sets generated and analysed during this study are available from the corresponding author on reasonable request.

## Electronic supplementary material

Supplementary Material
