# Supplementary material for: Determinants of initial inhaled corticosteroid use in patients with GOLD A/B COPD: a retrospective study of UK general practice
Source: NPJ Prim Care Respir Med. 2017 Jun 29;27:43. doi: 10.1038/s41533-017-0040-z (PMC5491501; doi:10.1038/s41533-017-0040-z)
Supplement: Supplementary file 1 — Supplementary Material [file 41533_2017_40_MOESM1_ESM.docx]

# Supplementary material

Table S1: Baseline patient characteristics (total patient cohort)

| **Characteristic** | **N** | **%** |
| --- | --- | --- |
| All patients | 29,815 | 100.0 |
| Gender (% male) | 16,057 | 53.9 |
| Age |  |  |
| 40-49 years | 1,977 | 6.6 |
| 50-59 years | 5,714 | 19.2 |
| 60-69 years | 10,065 | 33.8 |
| 70-79 years | 8,520 | 28.6 |
| 80-89 years | 3,358 | 11.3 |
| >90 years | 181 | 0.6 |
| Year of diagnosis |  |  |
| 2005 | 1,444 | 4.8 |
| 2006 | 3,301 | 11.1 |
| 2007 | 3,420 | 11.5 |
| 2008 | 3,382 | 11.3 |
| 2009 | 3,511 | 11.8 |
| 2010 | 3,406 | 11.4 |
| 2011 | 3,249 | 10.9 |
| 2012 | 3,019 | 10.1 |
| 2013 | 2,569 | 8.6 |
| 2014 | 2,019 | 6.8 |
| 2015 | 495 | 1.7 |
| Comorbidities |  |  |
| Asthma |  |  |
| Historic | 851 | 2.9 |
| Concurrent | 5,947 | 19.9 |
| Stroke | 905 | 3.0 |
| MI | 2,005 | 6.7 |
| Diabetes | 2,790 | 9.4 |
| Osteoporosis/osteopenia | 2,082 | 7.0 |
| Eosinophilia^†^ | 6,899 | 23.1 |
| Prior history of pneumonia | 1,033 | 3.5 |
| Smoking (current or ex)^‡^ | 24,873 | 83.4 |
| BMI |  |  |
| Missing | 195 | 0.7 |
| Underweight | 1,068 | 3.6 |
| Normal | 10,356 | 34.7 |
| Overweight | 10,455 | 35.1 |
| Obese | 7,741 | 26.0 |
| FEV_1_ % predicted |  |  |
| 80-100% (GOLD 1) | 10,986 | 36.8 |
| 50-80% (GOLD 2) | 18,829 | 63.2 |
| Moderate exacerbations^¶^ | 324 | 1.1 |
| UK region |  |  |
| North East | 674 | 2.3 |
| North West | 4522 | 15.2 |
| Yorkshire & The Humber | 811 | 2.7 |
| East Midlands | 853 | 2.9 |
| West Midlands | 2660 | 8.9 |
| East of England | 1919 | 6.4 |
| South West | 2456 | 8.2 |
| South Central | 3024 | 10.1 |
| London | 2633 | 8.8 |
| South East Central | 2616 | 8.8 |
| Northern Ireland | 1279 | 4.3 |
| Scotland | 3118 | 10.5 |
| Wales | 3250 | 10.9 |

BMI: body mass index; FEV_1_: forced expiratory volume in 1 second; GOLD: Global Initiative for Chronic Obstructive Lung Disease; MI: myocardial infarction.

† Eosinophilia was calculated using both clinical codes and test results for eosinophil count at a cut-off of >0.4 × 10^9^ / L

‡ Ex- and current smokers were pooled due to limitations in CPRD for distinguishing between these two groups

¶ In the year prior to COPD diagnosis (the index date).

Figure S1: Patients included in the study population


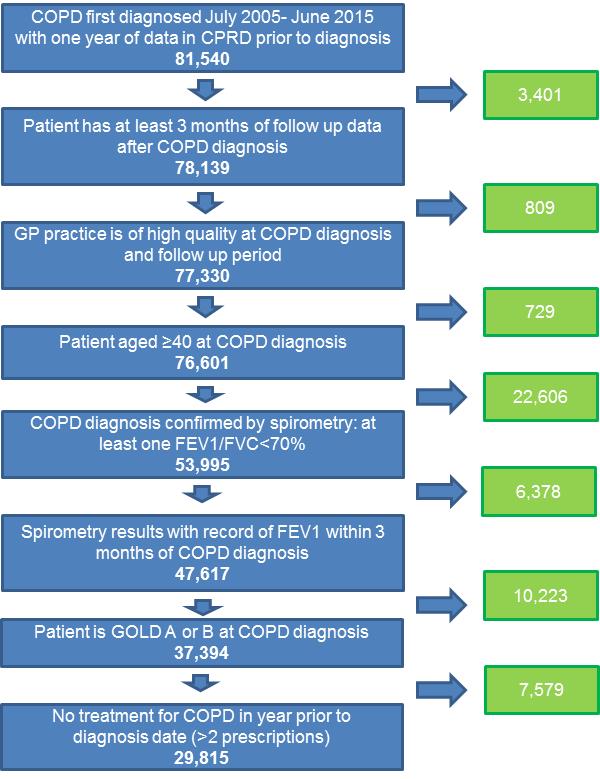


Figure S2: Number of patients by therapy combination at COPD diagnosis (overall cohort vs. cohort excluding patients with concurrent asthma)

COPD: chronic obstructive pulmonary disease; ICS: inhaled corticosteroids; SABA: short-acting beta agonists; SAMA: short-acting muscarinic antagonists.

Figure S3: Significant drivers of ICS prescription in patients with ACOS from logistic regression analysis


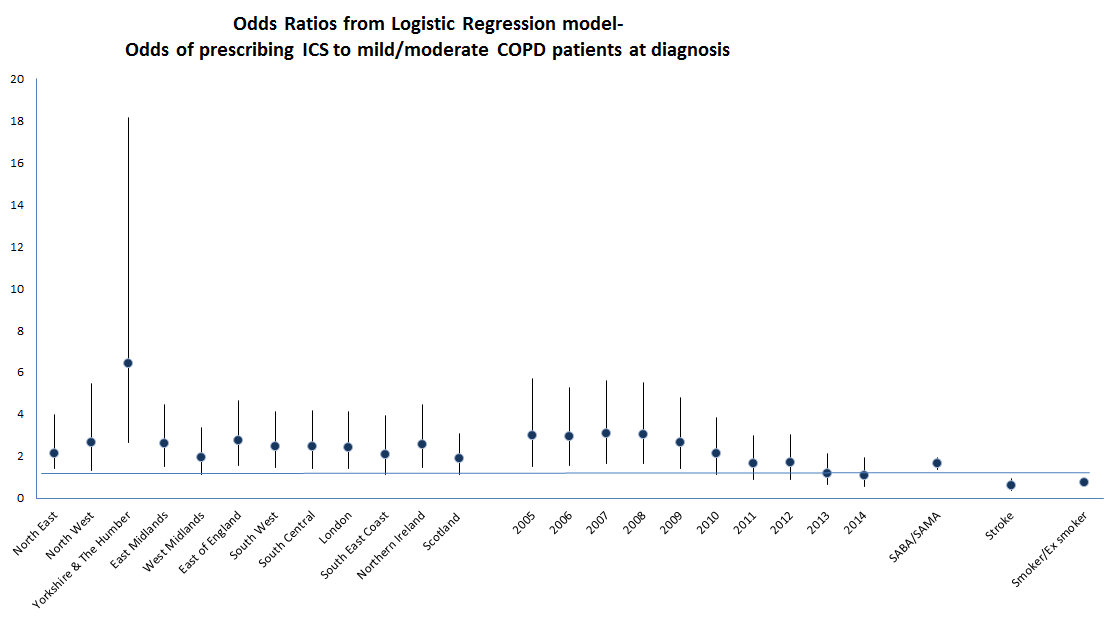


ACOS: asthma-COPD overlap syndrome; ICS: inhaled corticosteroids; SABA: short-acting beta agonists; SAMA: short-acting muscarinic antagonists.

Table S2: Study inclusion and exclusion criteria

| **Inclusion criteria** | **Exclusion criteria** |
| --- | --- |
| - Patient of acceptable quality standard in CPRD and alive and currently registered at a CPRD practice during the study period (June 2005 – June 2015) - A diagnosis of COPD after study start date (any recorded COPD diagnostic Read code plus spirometry confirming the diagnosis; individuals with any record of FEV_1_/FVC ratio <0.7^†^) - Aged ≥40 years at date of COPD diagnosis - At least one full year of data prior to index diagnosis - Patient in GOLD stage A or B (FEV_1_ ≥50% predicted using the record closest to COPD diagnosis and no more than 3 months after diagnosis. For patients with no FEV_1_ % predicted recorded, this was calculated using available FEV_1_ post-bronchodilation measures with either GP recorded expected FEV_1_ or estimated FEV_1_ using the ECCS equation.^(^[^1^](#_ENREF_1)^)^ Exacerbations were defined using Read codes for exacerbations; patients with 2 or more records in the year prior to diagnosis were excluded from the analysis.) | - Patients with treatment of interest in the year prior to diagnosis date - Patients with insufficient information in the CPRD record to confirm COPD using spirometry - Patients with insufficient information in their CPRD record to calculate baseline GOLD stage (no FEV_1_ % predicted record within 3 months of COPD diagnosis) |

COPD: chronic obstructive pulmonary disease; CPRD: Clinical Practice Research Datalink; ECCS: European Community of Coal and Steel; FEV_1_: forced expiratory volume in 1 second; GOLD: Global Initiative for Chronic Obstructive Lung Disease.

†FEV_1_/FVC was taken from GP recorded values, which do not specify if values are based on pre- or post-bronchodilator spirometry.

Table S3: Product codes for therapies of interest

| **Product code** | **Product Name** |
| --- | --- |
| **LAMA** | |
| 6050 | Spiriva 18 microgram Capsule (Boehringer Ingelheim Ltd) |
| 36869 | Spiriva Respimat 2.5micrograms/dose solution for inhalation cartridge with device (Boehringer Ingelheim Ltd) |
| 51967 | Spiriva 18microgram inhalation powder capsules (Mawdsley-Brooks & Company Ltd) |
| 35000 | Spiriva 18microgram inhalation powder capsules (Boehringer Ingelheim Ltd) |
| 34995 | Spiriva 18microgram inhalation powder capsules with HandiHaler (Boehringer Ingelheim Ltd) |
| 64232 | Tiotropium bromide 2.5micrograms/dose solution for inhalation cartridge with device CFC free (AM Distributions (Yorkshire) Ltd) |
| 35011 | Tiotropium bromide 18microgram inhalation powder capsules |
| 50577 | Spiriva 18microgram inhalation powder capsules with HandiHaler (DE Pharmaceuticals) |
| 36864 | Tiotropium bromide 2.5micrograms/dose solution for inhalation cartridge with device CFC free |
| 746 | Tiotropium 18 microgram Capsule |
| 35014 | Tiotropium bromide 18microgram inhalation powder capsules with device |
| 61582 | Spiriva Respimat 2.5micrograms/dose solution for inhalation cartridge with device (Waymade Healthcare Plc) |
| 49227 | Aclidinium bromide 375micrograms/dose dry powder inhaler |
| 49228 | Eklira 322micrograms/dose Genuair (AstraZeneca UK Ltd) |
| 63992 | Eklira 322micrograms/dose Genuair (Waymade Healthcare Plc) |
| 53982 | SeeBri Breezehaler 44microgram inhalation powder capsules |
| **LABA** | |
| 7268 | Serevent 25micrograms/dose Evohaler (GlaxoSmithKline UK Ltd) |
| 47638 | Neovent 25micrograms/dose inhaler CFC free (Kent Pharmaceuticals Ltd) |
| 35165 | Serevent 50microgram disks with Diskhaler (GlaxoSmithKline UK Ltd) |
| 7270 | Salmeterol 25micrograms/dose inhaler CFC free |
| 549 | Serevent 25micrograms/dose inhaler (GlaxoSmithKline UK Ltd) |
| 57694 | Vertine 25micrograms/dose inhaler CFC free (Teva UK Ltd) |
| 54742 | Salmeterol 25micrograms/dose inhaler CFC free (A A H Pharmaceuticals Ltd) |
| 50051 | Serevent 25micrograms/dose Evohaler (Waymade Healthcare Plc) |
| 35825 | Serevent 50microgram disks (GlaxoSmithKline UK Ltd) |
| 57544 | Serevent 50micrograms/dose Accuhaler (Waymade Healthcare Plc) |
| 719 | Salmeterol 50micrograms/dose dry powder inhaler |
| 465 | Salmeterol 25micrograms/dose inhaler |
| 35503 | Salmeterol 50microgram inhalation powder blisters |
| 35542 | Salmeterol 50microgram inhalation powder blisters with device |
| 56478 | Serevent 50micrograms/dose Accuhaler (DE Pharmaceuticals) |
| 3297 | Salmeterol 50micrograms disc |
| 910 | Serevent diskhaler 50microgram Inhalation powder (Glaxo Wellcome UK Ltd) |
| 2224 | Serevent 50micrograms/dose Accuhaler (GlaxoSmithKline UK Ltd) |
| 6526 | Formoterol 12microgram inhalation powder capsules with device |
| 1975 | Oxis 6 Turbohaler (AstraZeneca UK Ltd) |
| 35725 | Formoterol Easyhaler 12micrograms/dose dry powder inhaler (Orion Pharma (UK) Ltd) |
| 10968 | Foradil 12microgram inhalation powder capsules with device (Novartis Pharmaceuticals UK Ltd) |
| 57558 | Oxis 6 Turbohaler (Lexon (UK) Ltd) |
| 7133 | Formoterol 12micrograms/dose dry powder inhaler |
| 1974 | Oxis 12 Turbohaler (AstraZeneca UK Ltd) |
| 9711 | Formoterol 6micrograms/dose dry powder inhaler |
| 14306 | Formoterol 12micrograms/dose inhaler CFC free |
| 56482 | Oxis 12 Turbohaler (Waymade Healthcare Plc) |
| 25784 | Atimos Modulite 12micrograms/dose inhaler (Chiesi Ltd) |
| 44064 | Onbrez Breezhaler 300microgram inhalation powder capsules with device (Novartis Pharmaceuticals UK Ltd) |
| 43893 | Onbrez Breezhaler 150microgram inhalation powder capsules with device (Novartis Pharmaceuticals UK Ltd) |
| 43738 | Indacaterol 150microgram inhalation powder capsules with device |
| 45610 | Indacaterol 300microgram inhalation powder capsules with device |
| 62662 | Olodaterol 2.5micrograms/dose solution for inhalation cartridge with device CFC free |
| **ICS** | |
| 38 | Beclometasone 100micrograms/dose inhaler |
| 99 | Becotide 100 inhaler (GlaxoSmithKline UK Ltd) |
| 454 | Pulmicort 200microgram Inhaler (AstraZeneca UK Ltd) |
| 883 | Becodisks 200microgram Disc (Allen & Hanburys Ltd) |
| 895 | Beclazone 100 Easi-Breathe inhaler (Teva UK Ltd) |
| 896 | Becotide easi-breathe 100microgram/actuation Pressurised inhalation (Allen & Hanburys Ltd) |
| 908 | Pulmicort 400 Turbohaler (AstraZeneca UK Ltd) |
| 911 | Flixotide accuhaler 250 250microgram/inhalation Inhalation powder (Allen & Hanburys Ltd) |
| 947 | Budesonide 50micrograms/actuation refill canister |
| 956 | Pulmicort 200 Turbohaler (AstraZeneca UK Ltd) |
| 960 | Pulmicort 100 Turbohaler (AstraZeneca UK Ltd) |
| 1100 | Beclazone 100 inhaler (Teva UK Ltd) |
| 1236 | Becloforte 250micrograms/dose inhaler (GlaxoSmithKline UK Ltd) |
| 1242 | Beclometasone 250micrograms/dose inhaler |
| 1243 | Beclazone 250 Easi-Breathe inhaler (Teva UK Ltd) |
| 1258 | Becotide 200 inhaler (GlaxoSmithKline UK Ltd) |
| 1259 | Beclometasone 200micrograms/dose inhaler |
| 1269 | Becotide 50microgram/ml Nebuliser liquid (Allen & Hanburys Ltd) |
| 1406 | Becotide 50 inhaler (GlaxoSmithKline UK Ltd) |
| 1412 | Flixotide 250microgram/actuation Inhalation powder (Allen & Hanburys Ltd) |
| 1424 | Flixotide 250microgram Disc (Allen & Hanburys Ltd) |
| 1426 | Flixotide 500microgram Disc (Allen & Hanburys Ltd) |
| 1518 | Flixotide 50microgram/actuation Inhalation powder (Allen & Hanburys Ltd) |
| 1537 | Becotide 200microgram Rotacaps (GlaxoSmithKline UK Ltd) |
| 1551 | Beclazone 250 inhaler (Teva UK Ltd) |
| 1552 | Becloforte easi-breathe 250microgram/actuation Pressurised inhalation (Allen & Hanburys Lt |
| 1642 | Budesonide 400micrograms/dose dry powder inhaler |
| 1676 | Flixotide 125microgram/actuation Inhalation powder (Allen & Hanburys Ltd) |
| 1680 | Pulmicort LS 50micrograms/dose inhaler (AstraZeneca UK Ltd) |
| 1725 | Beclazone 50 Easi-Breathe inhaler (Teva UK Ltd) |
| 1727 | Becotide easi-breathe 50microgram/actuation Pressurised inhalation (Allen & Hanburys Ltd) |
| 1734 | Beclometasone 100micrograms/dose breath actuated inhaler |
| 1861 | AeroBec 100 Autohaler (Meda Pharmaceuticals Ltd) |
| 1885 | Beclazone 200 inhaler (Teva UK Ltd) |
| 1951 | Becodisks 400microgram Disc (Allen & Hanburys Ltd) |
| 1956 | Pulmicort 1mg Respules (AstraZeneca UK Ltd) |
| 1959 | Pulmicort 0.5mg Respules (AstraZeneca UK Ltd) |
| 2092 | Budesonide 200micrograms/dose dry powder inhaler |
| 2125 | Pulmicort 200microgram Refill canister (AstraZeneca UK Ltd) |
| 2148 | Beclometasone 400microgram disc |
| 2159 | AeroBec 50 Autohaler (Meda Pharmaceuticals Ltd) |
| 2160 | Beclometasone 50micrograms/dose breath actuated inhaler |
| 2229 | Becodisks 100microgram Disc (Allen & Hanburys Ltd) |
| 2282 | Fluticasone 500micrograms/dose dry powder inhaler |
| 2335 | Qvar 100 inhaler (Teva UK Ltd) |
| 2440 | Flixotide accuhaler 500 500microgram/inhalation Inhalation powder (Allen & Hanburys Ltd) |
| 2600 | Beclometasone 250micrograms/dose breath actuated inhaler |
| 2723 | Fluticasone 25micrograms/dose inhaler |
| 2892 | Becloforte 400microgram disks (GlaxoSmithKline UK Ltd) |
| 2893 | Beclometasone 200micrograms disc |
| 2951 | Fluticasone 250microgram/actuation Pressurised inhalation |
| 2992 | Beclazone 50 inhaler (Teva UK Ltd) |
| 3018 | Beclometasone 50micrograms/dose inhaler |
| 3075 | Becotide 400microgram Rotacaps (GlaxoSmithKline UK Ltd) |
| 3119 | Becloforte integra 250microgram/actuation Inhaler with compact spacer (Glaxo Laboratories |
| 3150 | Beclometasone 100micrograms/actuation extrafine particle cfc free inhaler |
| 3220 | Qvar 50 Autohaler (Teva UK Ltd) |
| 3289 | Flixotide 25micrograms/dose inhaler (GlaxoSmithKline UK Ltd) |
| 3363 | Becloforte 400microgram disks with Diskhaler (GlaxoSmithKline UK Ltd) |
| 3546 | Qvar 50 inhaler (Teva UK Ltd) |
| 3570 | Budesonide 200micrograms/actuation refill canister |
| 3743 | Filair 50 inhaler (Meda Pharmaceuticals Ltd) |
| 3927 | Filair 100 inhaler (Meda Pharmaceuticals Ltd) |
| 3947 | Becotide 100microgram Rotacaps (GlaxoSmithKline UK Ltd) |
| 3989 | Flixotide 100microgram Disc (Allen & Hanburys Ltd) |
| 3993 | Filair Forte 250micrograms/dose inhaler (Meda Pharmaceuticals Ltd) |
| 4131 | Fluticasone 100microgram Disc |
| 4132 | Fluticasone 125microgram/actuation Pressurised inhalation |
| 4365 | Beclometasone 100micrograms disc |
| 4413 | Qvar 100 Autohaler (Teva UK Ltd) |
| 4499 | Aerobec 250microgram/actuation Pressurised inhalation (Meda Pharmaceuticals Ltd) |
| 4545 | Pulmicort LS 50microgram Refill canister (AstraZeneca UK Ltd) |
| 4601 | Asmabec 100 Clickhaler (Focus Pharmaceuticals Ltd) |
| 4688 | Fluticasone 50microgram/actuation Pressurised inhalation |
| 4759 | Beclometasone 100microgram inhalation powder capsules |
| 4801 | Budesonide 500micrograms/2ml nebuliser liquid unit dose vials |
| 4803 | Beclazone 250microgram/actuation Inhalation powder (Actavis UK Ltd) |
| 4926 | Flixotide accuhaler 100 100microgram/inhalation Inhalation powder (Allen & Hanburys Ltd) |
| 4942 | Budesonide 1mg/2ml nebuliser liquid unit dose vials |
| 5223 | Fluticasone 50micrograms/dose inhaler CFC free |
| 5309 | Flixotide 50micrograms/dose Evohaler (GlaxoSmithKline UK Ltd) |
| 5521 | Beclometasone 200micrograms/dose dry powder inhaler |
| 5522 | Beclometasone 100micrograms/dose dry powder inhaler |
| 5551 | Flixotide 0.5mg/2ml Nebules (GlaxoSmithKline UK Ltd) |
| 5580 | Flixotide accuhaler 50 50microgram/inhalation Inhalation powder (Allen & Hanburys Ltd) |
| 5683 | Flixotide 250micrograms/dose Evohaler (GlaxoSmithKline UK Ltd) |
| 5718 | Flixotide 125micrograms/dose Evohaler (GlaxoSmithKline UK Ltd) |
| 5804 | Beclometasone 250micrograms/dose dry powder inhaler |
| 5822 | Fluticasone 250micrograms/dose inhaler CFC free |
| 5885 | Fluticasone 100micrograms/dose dry powder inhaler |
| 5975 | Fluticasone 125micrograms/dose inhaler CFC free |
| 5992 | Beclometasone 50micrograms/dose dry powder inhaler |
| 6839 | Alvesco 160 inhaler (Takeda UK Ltd) |
| 7356 | Ciclesonide 80micrograms/dose inhaler CFC free |
| 7602 | Fluticasone 50microgram Disc |
| 7638 | Fluticasone 250microgram Disc |
| 7653 | Beclometasone 400microgram inhalation powder capsules |
| 7788 | Budesonide 100micrograms/dose dry powder inhaler |
| 7891 | Fluticasone 500microgram Disc |
| 7948 | Fluticasone 250micrograms/dose dry powder inhaler |
| 7964 | Beclometasone 50micrograms/ml nebuliser suspension |
| 8111 | Becloforte vm 250microgram/actuation VM pack (Allen & Hanburys Ltd) |
| 8433 | Budesonide 100micrograms/actuation inhaler |
| 8635 | Flixotide 50microgram Disc (Allen & Hanburys Ltd) |
| 9164 | Fluticasone 50micrograms/dose dry powder inhaler |
| 9233 | Beclometasone 200microgram inhalation powder capsules |
| 9477 | Asmabec 100microgram/actuation Spacehaler (Celltech Pharma Europe Ltd) |
| 9571 | Beclometasone 250micrograms/actuation vortex inhaler |
| 9577 | Asmabec 50 Clickhaler (Focus Pharmaceuticals Ltd) |
| 9599 | Beclazone 50microgram/actuation Inhalation powder (Actavis UK Ltd) |
| 9921 | Beclometasone 100micrograms/dose breath actuated inhaler CFC free |
| 10090 | Beclometasone 50micrograms/actuation extrafine particle cfc free inhaler |
| 10102 | Ciclesonide 160micrograms/dose inhaler CFC free |
| 10254 | Mometasone 400micrograms/dose dry powder inhaler |
| 11198 | Beclometasons 50 micrograms/actuation vortex inhaler |
| 11478 | Fluticasone 2mg/2ml nebuliser liquid unit dose vials |
| 11497 | Beclometasone 400micrograms/dose dry powder inhaler |
| 11732 | Beclometasone 50micrograms/dose breath actuated inhaler CFC free |
| 13037 | Pulvinal Beclometasone Dipropionate 200micrograms/dose dry powder inhaler (Chiesi Ltd) |
| 13290 | Clenil Modulite 100micrograms/dose inhaler (Chiesi Ltd) |
| 13815 | Beclazone 100microgram/actuation Inhalation powder (Actavis UK Ltd) |
| 14294 | Qvar 50micrograms/dose Easi-Breathe inhaler (Teva UK Ltd) |
| 14321 | Beclometasone 200micrograms/dose inhaler CFC free |
| 14524 | Bdp 250microgram/actuation Spacehaler (Celltech Pharma Europe Ltd) |
| 14567 | Asmabec 250 Clickhaler (Focus Pharmaceuticals Ltd) |
| 14590 | Asmabec 250microgram/actuation Spacehaler (Celltech Pharma Europe Ltd) |
| 14700 | Budesonide 400micrograms/actuation inhaler |
| 14736 | Pulvinal Beclometasone Dipropionate 400micrograms/dose dry powder inhaler (Chiesi Ltd) |
| 14757 | Pulvinal Beclometasone Dipropionate 100micrograms/dose dry powder inhaler (Chiesi Ltd) |
| 15326 | Beclometasone 100micrograms/dose inhaler CFC free |
| 15706 | Beclometasone 100 micrograms/actuation vortex inhaler |
| 16018 | Mometasone 200micrograms/dose dry powder inhaler |
| 16054 | Budesonide 200micrograms/actuation breath actuated powder inhaler |
| 16148 | Clenil Modulite 250micrograms/dose inhaler (Chiesi Ltd) |
| 16151 | Clenil Modulite 200micrograms/dose inhaler (Chiesi Ltd) |
| 16158 | Clenil Modulite 50micrograms/dose inhaler (Chiesi Ltd) |
| 16305 | Flixotide 2mg/2ml Nebules (GlaxoSmithKline UK Ltd) |
| 16433 | Asmanex 200micrograms/dose Twisthaler (Merck Sharp & Dohme Ltd) |
| 16584 | Beclometasone 50micrograms/dose inhaler CFC free |
| 17465 | Fluticasone 500micrograms/2ml nebuliser liquid unit dose vials |
| 17590 | Asmanex 400micrograms/dose Twisthaler (Merck Sharp & Dohme Ltd) |
| 17654 | Easyhaler Beclometasone 200micrograms/dose dry powder inhaler (Orion Pharma (UK) Ltd) |
| 17670 | Easyhaler Budesonide 100micrograms/dose dry powder inhaler (Orion Pharma (UK) Ltd) |
| 18394 | Bdp 50microgram/actuation Spacehaler (Celltech Pharma Europe Ltd) |
| 18848 | Qvar 100micrograms/dose Easi-Breathe inhaler (Teva UK Ltd) |
| 19031 | Bdp 100microgram/actuation Spacehaler (Celltech Pharma Europe Ltd) |
| 19389 | Asmabec 50microgram/actuation Spacehaler (Celltech Pharma Europe Ltd) |
| 19401 | Beclometasone 250micrograms/actuation inhaler and compact spacer |
| 20825 | Spacehaler BDP 250microgram/actuation Spacehaler (Celltech Pharma Europe Ltd) |
| 21005 | Beclometasone 250micrograms/dose inhaler CFC free |
| 21224 | Alvesco 80 inhaler (Takeda UK Ltd) |
| 21482 | Beclometasone 100micrograms/dose inhaler (Generics (UK) Ltd) |
| 23741 | Novolizer budesonide 200microgram/actuation Pressurised inhalation (Meda Pharmaceuticals L |
| 24898 | Spacehaler BDP 100microgram/actuation Spacehaler (Celltech Pharma Europe Ltd) |
| 25204 | Beclometasone 100micrograms/dose inhaler (A A H Pharmaceuticals Ltd) |
| 26063 | Beclometasone 100micrograms/dose inhaler (Teva UK Ltd) |
| 27188 | Easyhaler Budesonide 200micrograms/dose dry powder inhaler (Orion Pharma (UK) Ltd) |
| 27679 | Beclometasone 100microgram/actuation Pressurised inhalation (Approved Prescription Service |
| 28073 | Beclometasone 250microgram/actuation Pressurised inhalation (Approved Prescription Service |
| 28640 | Beclometasone 100microgram/actuation Inhalation powder (Actavis UK Ltd) |
| 28761 | Spacehaler BDP 50microgram/actuation Spacehaler (Celltech Pharma Europe Ltd) |
| 29325 | Beclometasone 250micrograms/dose inhaler (Generics (UK) Ltd) |
| 30210 | Beclometasone 250micrograms/dose inhaler (Teva UK Ltd) |
| 30238 | Beclometasone 50microgram/actuation Pressurised inhalation (Approved Prescription Services |
| 30649 | Easyhaler Budesonide 400micrograms/dose dry powder inhaler (Orion Pharma (UK) Ltd) |
| 31774 | Beclometasone 50micrograms/dose inhaler (Generics (UK) Ltd) |
| 32874 | Beclometasone 50microgram/actuation Inhalation powder (Actavis UK Ltd) |
| 33258 | Beclometasone 250micrograms/dose inhaler (A A H Pharmaceuticals Ltd) |
| 33849 | Beclometasone 100microgram/actuation Inhalation powder (Neo Laboratories Ltd) |
| 34315 | Beclometasone 250microgram/actuation Inhalation powder (Actavis UK Ltd) |
| 34428 | Beclometasone 50microgram/actuation Inhalation powder (Neo Laboratories Ltd) |
| 34739 | Beclometasone 50micrograms/dose inhaler (Teva UK Ltd) |
| 34794 | Beclometasone 200micrograms/dose inhaler (A A H Pharmaceuticals Ltd) |
| 34859 | Beclometasone 250microgram/actuation Inhalation powder (Neo Laboratories Ltd) |
| 34919 | Beclometasone 50micrograms/dose inhaler (A A H Pharmaceuticals Ltd) |
| 35071 | Becodisks 200microgram (GlaxoSmithKline UK Ltd) |
| 35106 | Becodisks 100microgram with Diskhaler (GlaxoSmithKline UK Ltd) |
| 35107 | Beclometasone 400microgram inhalation powder blisters with device |
| 35113 | Beclometasone 200microgram inhalation powder blisters |
| 35118 | Becodisks 400microgram with Diskhaler (GlaxoSmithKline UK Ltd) |
| 35225 | Flixotide 100microgram disks with Diskhaler (GlaxoSmithKline UK Ltd) |
| 35288 | Beclometasone 400microgram inhalation powder blisters |
| 35293 | Beclometasone 200microgram inhalation powder blisters with device |
| 35299 | Becodisks 400microgram (GlaxoSmithKline UK Ltd) |
| 35374 | Flixotide 500microgram disks (GlaxoSmithKline UK Ltd) |
| 35392 | Flixotide 500microgram disks with Diskhaler (GlaxoSmithKline UK Ltd) |
| 35408 | Becodisks 100microgram (GlaxoSmithKline UK Ltd) |
| 35430 | Becodisks 200microgram with Diskhaler (GlaxoSmithKline UK Ltd) |
| 35461 | Flixotide 250microgram disks with Diskhaler (GlaxoSmithKline UK Ltd) |
| 35510 | Budesonide 200micrograms/dose dry powder inhalation cartridge with device |
| 35580 | Beclometasone 100microgram inhalation powder blisters with device |
| 35602 | Budesonide 200micrograms/dose dry powder inhalation cartridge |
| 35611 | Flixotide 250microgram disks (GlaxoSmithKline UK Ltd) |
| 35631 | Budelin Novolizer 200micrograms/dose inhalation powder (Meda Pharmaceuticals Ltd) |
| 35638 | Fluticasone 100microgram inhalation powder blisters with device |
| 35652 | Beclometasone 100microgram inhalation powder blisters |
| 35700 | Fluticasone 500microgram inhalation powder blisters with device |
| 35724 | Budelin Novolizer 200micrograms/dose inhalation powder refill (Meda Pharmaceuticals Ltd) |
| 35772 | Fluticasone 100microgram inhalation powder blisters |
| 35905 | Fluticasone 250microgram inhalation powder blisters |
| 35986 | Flixotide 50microgram disks (GlaxoSmithKline UK Ltd) |
| 36021 | Fluticasone 50microgram inhalation powder blisters with device |
| 36090 | Flixotide 100microgram disks (GlaxoSmithKline UK Ltd) |
| 36290 | Flixotide 50microgram disks with Diskhaler (GlaxoSmithKline UK Ltd) |
| 36401 | Fluticasone 250microgram inhalation powder blisters with device |
| 36462 | Fluticasone 500microgram inhalation powder blisters |
| 37447 | Fluticasone 50microgram inhalation powder blisters |
| 39200 | AeroBec Forte 250 Autohaler (Meda Pharmaceuticals Ltd) |
| 40057 | Pulmicort 200micrograms/dose inhaler CFC free (AstraZeneca UK Ltd) |
| 41269 | Beclometasone 400 Cyclocaps (Teva UK Ltd) |
| 41412 | Beclometasone 400micrograms/actuation inhaler |
| 42928 | Flixotide 100micrograms/dose Accuhaler (GlaxoSmithKline UK Ltd) |
| 42985 | Flixotide 50micrograms/dose Accuhaler (GlaxoSmithKline UK Ltd) |
| 42994 | Flixotide 250micrograms/dose Accuhaler (GlaxoSmithKline UK Ltd) |
| 43074 | Flixotide 500micrograms/dose Accuhaler (GlaxoSmithKline UK Ltd) |
| 46157 | Beclometasone 200 Cyclocaps (Teva UK Ltd) |
| 47943 | Beclazone easi-breathe (roi) 100microgram/actuation Pressurised inhalation (Ivax Pharmaceu |
| 48340 | Clenil Modulite 100micrograms/dose inhaler (Mawdsley-Brooks & Company Ltd) |
| 48709 | Qvar 100micrograms/dose Easi-Breathe inhaler (Sigma Pharmaceuticals Plc) |
| 49367 | Clenil Modulite 50micrograms/dose inhaler (Mawdsley-Brooks & Company Ltd) |
| 49412 | Clenil Modulite 200micrograms/dose inhaler (Mawdsley-Brooks & Company Ltd) |
| 49711 | Pulmicort 200micrograms/dose inhaler (AstraZeneca UK Ltd) |
| 49772 | Fluticasone 250micrograms/dose Evohaler (Sigma Pharmaceuticals Plc) |
| 50037 | Pulmicort 0.5mg Respules (Waymade Healthcare Plc) |
| 50129 | Qvar 100micrograms/dose Easi-Breathe inhaler (Doncaster Pharmaceuticals Ltd) |
| 50287 | Qvar 100 inhaler (Doncaster Pharmaceuticals Ltd) |
| 51234 | Qvar 100 inhaler (Waymade Healthcare Plc) |
| 51415 | Qvar 50 inhaler (Mawdsley-Brooks & Company Ltd) |
| 51480 | Qvar 100 Autohaler (Doncaster Pharmaceuticals Ltd) |
| 51681 | Qvar 100 inhaler (Sigma Pharmaceuticals Plc) |
| 51815 | Flixotide 250micrograms/dose Evohaler (Waymade Healthcare Plc) |
| 52732 | Pulmicort 0.5mg Respules (Necessity Supplies Ltd) |
| 52806 | Qvar 100 Autohaler (Lexon (UK) Ltd) |
| 53057 | Flixotide 50micrograms/dose Evohaler (Lexon (UK) Ltd) |
| 53480 | Qvar 100 Autohaler (Stephar (U.K.) Ltd) |
| 54207 | Qvar 50 inhaler (Doncaster Pharmaceuticals Ltd) |
| 54399 | Qvar 100 Autohaler (Sigma Pharmaceuticals Plc) |
| 56462 | Becodisks 400microgram (Waymade Healthcare Plc) |
| 56471 | Becodisks 200microgram (Mawdsley-Brooks & Company Ltd) |
| 56474 | Flixotide 125micrograms/dose Evohaler (Doncaster Pharmaceuticals Ltd) |
| 56475 | Flixotide 50micrograms/dose Accuhaler (Sigma Pharmaceuticals Plc) |
| 56477 | Flixotide 100micrograms/dose Accuhaler (Waymade Healthcare Plc) |
| 56484 | Flixotide 250micrograms/dose Accuhaler (Waymade Healthcare Plc) |
| 56493 | Qvar 50micrograms/dose Easi-Breathe inhaler (Sigma Pharmaceuticals Plc) |
| 56498 | Pulmicort 200 Turbohaler (Waymade Healthcare Plc) |
| 56499 | Flixotide 500micrograms/dose Accuhaler (Waymade Healthcare Plc) |
| 57525 | Flixotide 250micrograms/dose Accuhaler (Stephar (U.K.) Ltd) |
| 57555 | Flixotide 125micrograms/dose Evohaler (Dowelhurst Ltd) |
| 57579 | Flixotide 50micrograms/dose Accuhaler (Doncaster Pharmaceuticals Ltd) |
| 57589 | Becloforte 250micrograms/dose inhaler (Dowelhurst Ltd) |
| 61664 | Clenil Modulite 250micrograms/dose inhaler (Waymade Healthcare Plc) |
| 60937 | Pulmicort 200 Turbohaler (Dowelhurst Ltd) |
| 61975 | Budesonide 500micrograms/2ml nebuliser liquid unit dose vials (Almus Pharmaceuticals Ltd) |
| 62341 | Becotide 50 inhaler (Dowelhurst Ltd) |
| 62518 | Beclometasone 100micrograms/dose inhaler CFC free (Ennogen Healthcare Ltd) |
| 63585 | Beclometasone 50micrograms/dose inhaler (Almus Pharmaceuticals Ltd) |
| 61664 | Clenil Modulite 250micrograms/dose inhaler (Waymade Healthcare Plc) |
| **ICS + LABA** | |
| 638 | Seretide 250 Accuhaler (GlaxoSmithKline UK Ltd) |
| 665 | Seretide 100 Accuhaler (GlaxoSmithKline UK Ltd) |
| 3666 | Seretide 500 Accuhaler (GlaxoSmithKline UK Ltd) |
| 5143 | Seretide 50 Evohaler (GlaxoSmithKline UK Ltd) |
| 5161 | Seretide 125 Evohaler (GlaxoSmithKline UK Ltd) |
| 5172 | Seretide 250 Evohaler (GlaxoSmithKline UK Ltd) |
| 5558 | Salmeterol 50micrograms with fluticasone 500micrograms CFC free inhaler |
| 5864 | Salmeterol 25micrograms with fluticasone 250micrograms CFC free inhaler |
| 5942 | Salmeterol 50micrograms with fluticasone 250micrograms CFC free inhaler |
| 6325 | Symbicort 200/6 Turbohaler (AstraZeneca UK Ltd) |
| 6569 | Salmeterol 25micrograms with fluticasone 125micrograms CFC free inhaler |
| 6616 | Salmeterol 25micrograms with fluticasone 50micrograms CFC free inhaler |
| 6746 | Budenoside 400micrograms/dose / Formoterol 12 micrograms/dose dry powder inhaler |
| 6780 | Symbicort 400/12 Turbohaler (AstraZeneca UK Ltd) |
| 6796 | Budesonide 200micrograms/dose / Formoterol 6micrograms/dose dry powder inhaler |
| 6938 | Salmeterol 50micrograms with fluticasone 100micrograms CFC free inhaler |
| 7013 | Symbicort 100/6 Turbohaler (AstraZeneca UK Ltd) |
| 10218 | Budesonide 100micrograms/dose / Formoterol 6micrograms/dose dry powder inhaler |
| 11410 | Fluticasone 500micrograms/dose / Salmeterol 50micrograms/dose dry powder inhal |
| 11588 | Fluticasone 125micrograms/dose / Salmeterol 25micrograms/dose inhaler CFC free |
| 11618 | Fluticasone 250micrograms/dose / Salmeterol 25micrograms/dose inhaler CFC free |
| 12994 | Fluticasone 50micrograms/dose / Salmeterol 25micrograms/dose inhaler CFC free |
| 13040 | Fluticasone 250micrograms/dose / Salmeterol 50micrograms/dose dry powder inhal |
| 13273 | Fluticasone 100micrograms/dose / Salmeterol 50micrograms/dose dry powder inhal |
| 37432 | Fostair 100micrograms/dose/6micrograms/dose inhaler (Chiesi Ltd) |
| 37470 | Beclometasone 100micrograms/dose / Formoterol 6micrograms/dose inhaler CFC fre |
| 48739 | Seretide 250 Evohaler (Doncaster Pharmaceuticals Ltd) |
| 49000 | Seretide 250 Evohaler (Waymade Healthcare Plc) |
| 49114 | Symbicort 100/6 Turbohaler (Sigma Pharmaceuticals Plc) |
| 50560 | Seretide 250 Accuhaler (Sigma Pharmaceuticals Plc) |
| 50739 | Symbicort 400/12 Turbohaler (Mawdsley-Brooks & Company Ltd) |
| 50886 | Seretide 250 Evohaler (Stephar (U.K.) Ltd) |
| 50945 | Symbicort 100/6 Turbohaler (Mawdsley-Brooks & Company Ltd) |
| 51027 | Seretide 125 Evohaler (Doncaster Pharmaceuticals Ltd) |
| 51151 | Seretide 125 Evohaler (Lexon (UK) Ltd) |
| 51394 | Seretide 500 Accuhaler (Waymade Healthcare Plc) |
| 51570 | Symbicort 200/6 Turbohaler (Doncaster Pharmaceuticals Ltd) |
| 51593 | Seretide 500 Accuhaler (Doncaster Pharmaceuticals Ltd) |
| 51759 | Symbicort 200/6 Turbohaler (Mawdsley-Brooks & Company Ltd) |
| 51861 | Seretide 500 Accuhaler (Mawdsley-Brooks & Company Ltd) |
| 51909 | Seretide 250 Evohaler (Necessity Supplies Ltd) |
| 53230 | Seretide 250 Accuhaler (Doncaster Pharmaceuticals Ltd) |
| 53237 | Symbicort 400/12 Turbohaler (Doncaster Pharmaceuticals Ltd) |
| 53283 | Seretide 100 Accuhaler (Waymade Healthcare Plc) |
| 53491 | Symbicort 200/6 Turbohaler (Sigma Pharmaceuticals Plc) |
| 55677 | Seretide 500 Accuhaler (Lexon (UK) Ltd) |
| 61280 | Seretide 250 Accuhaler (Waymade Healthcare Plc) |
| 61666 | DuoResp Spiromax 320micrograms/dose / 9micrograms/dose dry powder inhaler (Teva UK Ltd) |
| 61782 | DuoResp Spiromax 160micrograms/dose / 4.5micrograms/dose dry powder inhaler (Teva UK Ltd) |
| 62126 | Seretide 100 Accuhaler (DE Pharmaceuticals) |
| 63252 | Seretide 250 Evohaler (Lexon (UK) Ltd) |
| 63945 | Seretide 250 Accuhaler (Lexon (UK) Ltd) |
| 64372 | Sirdupla 25micrograms/dose / 125micrograms/dose inhaler (Mylan Ltd) |
| 64373 | Sirdupla 25micrograms/dose / 250micrograms/dose inhaler (Mylan Ltd) |
| **LAMA + LABA** | |
| 62667 | Ultibro Breezhaler 85microgram/43microgram inhalation powder capsules with device (Novartis Pharmaceuticals UK Ltd) |
| 62739 | Indacaterol 85micrograms/dose / Glycopyrronium bromide 54micrograms/dose inhalation powder capsules with device |
| 64523 | Spiolto Respimat 2.5micrograms/dose / 2.5micrograms/dose solution for inhalation cartridge with device (Boehringer Ingelheim Ltd) |
| 64509 | Tiotropium bromide 2.5micrograms/dose / Olodaterol 2.5micrograms/dose solution for inhalation cartridge with device CFC free |
| 62838 | Aclidinium bromide 396micrograms/dose / Formoterol 11.8micrograms/dose dry powder inhaler |
| 62535 | Duaklir 340micrograms/dose / 12micrograms/dose Genuair (AstraZeneca UK Ltd) |
| 61176 | Anoro Ellipta 55micrograms/dose / 22micrograms/dose dry powder inhaler (GlaxoSmithKline UK Ltd) |
| **SABA/SAMA** | |
| 11046 | Ipratropium bromide with salbutamol 500micrograms + 2.5mg/2.5ml |
| 8 | Salbutamol 100micrograms/dose inhaler |
| 17 | Salbutamol 100micrograms/dose inhaler CFC free |
| 31 | Ventolin 100microgram/inhalation Inhalation powder (Glaxo Wellcome UK Ltd) |
| 235 | Bricanyl 250micrograms/dose inhaler (AstraZeneca UK Ltd) |
| 282 | Salbutamol 2mg/5ml oral solution sugar free |
| 510 | Ventolin 5mg/ml respirator solution (GlaxoSmithKline UK Ltd) |
| 534 | Atrovent 20micrograms/dose inhaler (Boehringer Ingelheim Ltd) |
| 556 | Combivent inhaler (Boehringer Ingelheim Ltd) |
| 674 | Ventolin 2.5mg Nebules (GlaxoSmithKline UK Ltd) |
| 696 | Salbutamol 8mg modified-release capsules |
| 856 | Ventolin 2mg/5ml syrup (GlaxoSmithKline UK Ltd) |
| 860 | Salbutamol 4mg tablets |
| 862 | Salbulin Inhalation powder (3M Health Care Ltd) |
| 881 | Salbutamol 2mg tablets |
| 882 | Salbutamol 200microgram inhalation powder capsules |
| 898 | Ventolin evohaler 100 100microgram/inhalation Pressurised inhalation (Glaxo Wellcome UK Ltd) |
| 907 | Bricanyl turbohaler 500 500microgram Turbohaler (AstraZeneca UK Ltd) |
| 942 | Aerolin 100micrograms/dose Autohaler (3M Health Care Ltd) |
| 957 | Salamol easi-breathe 100microgram/actuation Pressurised inhalation (IVAX Pharmaceuticals UK Ltd) |
| 958 | Ventolin easi-breathe 100microgram/actuation Pressurised inhalation (Allen & Hanburys Ltd) |
| 987 | Ventolin 4mg Tablet (Allen & Hanburys Ltd) |
| 1087 | Asmasal 95micrograms/dose Clickhaler (Focus Pharmaceuticals Ltd) |
| 1093 | Salamol 100microgram/actuation Inhalation powder (IVAX Pharmaceuticals UK Ltd) |
| 1346 | Salbutamol 0.05mg/ml injection |
| 1409 | Ipratropium bromide 20micrograms/dose inhaler |
| 1410 | Ipratropium bromide 0.25mg/ml |
| 1411 | Ipratropium bromide 250micrograms/ml |
| 1414 | Salamol 5mg/2.5ml nebuliser liquid Steri-Neb unit dose vials (Teva UK Ltd) |
| 1415 | Steri-neb ipratropium 250microgram/ml Nebuliser liquid (IVAX Pharmaceuticals UK Ltd) |
| 1619 | Terbutaline 500micrograms/dose dry powder inhaler |
| 1620 | Terbutaline 250micrograms/dose inhaler |
| 1628 | Terbutaline 250micrograms/actuation refill canister |
| 1630 | Salbutamol 2.5mg/2.5ml nebuliser liquid unit dose vials |
| 1635 | Salbuvent 2mg/5ml Oral solution (Pharmacia Ltd) |
| 1697 | Atrovent 20micrograms/dose Autohaler (Boehringer Ingelheim Ltd) |
| 1698 | Salbutamol 100micrograms/dose breath actuated inhaler |
| 1711 | Salbutamol 5mg/2.5ml nebuliser liquid unit dose vials |
| 1741 | Salbutamol 100micrograms/dose breath actuated inhaler CFC free |
| 1787 | Rinatec 21micrograms/dose nasal spray (Boehringer Ingelheim Ltd) |
| 1801 | Ventide inhaler (GlaxoSmithKline UK Ltd) |
| 1882 | Ventodisks 200microgram/blister Disc (Allen & Hanburys Ltd) |
| 1950 | Ventodisks 400microgram/blister Disc (Allen & Hanburys Ltd) |
| 1952 | Ventolin 400microgram Rotacaps (GlaxoSmithKline UK Ltd) |
| 1957 | Ventolin 5mg Nebules (GlaxoSmithKline UK Ltd) |
| 1960 | Volmax 8mg modified-release tablets (GlaxoSmithKline UK Ltd) |
| 1961 | Volmax 4mg modified-release tablets (GlaxoSmithKline UK Ltd) |
| 1962 | Atrovent udv 0.25mg/ml Nebuliser liquid (Boehringer Ingelheim Ltd) |
| 2097 | Ipratropium bromide 20micrograms/metered dose |
| 2099 | Rinatec 20microgram/metered dose Nasal spray (Boehringer Ingelheim Ltd) |
| 2152 | Ipratropium bromide with salbutamol 20mcg + 100mcg |
| 2655 | Airomir 100micrograms/dose inhaler (Teva UK Ltd) |
| 2722 | Duovent inhaler (Boehringer Ingelheim Ltd) |
| 2758 | Bricanyl Refill canister (AstraZeneca UK Ltd) |
| 2850 | Salbutamol 400microgram inhalation powder capsules |
| 2851 | Ventolin 200microgram Rotacaps (GlaxoSmithKline UK Ltd) |
| 2862 | Duovent Autohaler (Boehringer Ingelheim Ltd) |
| 2869 | Salbutamol 8mg modified-release tablets |
| 2978 | Salbutamol 200micrograms/dose dry powder inhaler |
| 2994 | Atrovent aerocaps 40microgram Inhalation powder (Boehringer Ingelheim Ltd) |
| 3163 | Salbutamol 200micrograms disc |
| 3254 | Salbulin 4mg Tablet (3M Health Care Ltd) |
| 3305 | Combivent nebuliser liquid 2.5ml UDVs (Boehringer Ingelheim Ltd) |
| 3306 | Atrovent Forte 40micrograms/dose inhaler (Boehringer Ingelheim Ltd) |
| 3443 | Salbutamol 100microgram/inhalation Spacehaler (Celltech Pharma Europe Ltd) |
| 3534 | Bricanyl 5mg tablets (AstraZeneca UK Ltd) |
| 3556 | Beclometasone 50micrograms with salbutamol 100micrograms/inhalation inhaler |
| 3584 | Bricanyl 1.5mg/5ml syrup (AstraZeneca UK Ltd) |
| 3786 | Fenoterol 100micrograms/dose / Ipratropium 40micrograms/dose inhaler |
| 3994 | Salbutamol 4mg modified-release tablets |
| 4055 | Salbulin 2mg/5ml Oral solution (3M Health Care Ltd) |
| 4171 | Ventolin 2mg Tablet (Allen & Hanburys Ltd) |
| 4222 | Bricanyl 10mg/ml respirator solution (AstraZeneca UK Ltd) |
| 4268 | Ipratropium bromide 40micrograms/dose inhaler |
| 4497 | Ventolin accuhaler 200 200microgram/actuation Inhalation powder (Glaxo Wellcome UK Ltd) |
| 4541 | Bricanyl SA 7.5mg tablets (AstraZeneca UK Ltd) |
| 4634 | Salamol 2.5mg/2.5ml nebuliser liquid Steri-Neb unit dose vials (Teva UK Ltd) |
| 4639 | Ipratropium bromide 21micrograms/dose nasal spray |
| 4640 | Bricanyl 5mg/2ml Nebuliser liquid (AstraZeneca UK Ltd) |
| 4665 | Salbulin 100micrograms/dose inhaler (3M Health Care Ltd) |
| 5170 | Salamol 100micrograms/dose inhaler CFC free (Teva UK Ltd) |
| 5308 | Terbutaline 5mg/2ml nebuliser liquid unit dose vials |
| 5516 | Salamol 100micrograms/dose Easi-Breathe inhaler (Teva UK Ltd) |
| 5740 | Airomir 100micrograms/dose Autohaler (Teva UK Ltd) |
| 5753 | Salbutamol 400micrograms disc |
| 5837 | Salamol steri-neb 5mg/2.5ml Nebuliser liquid (Numark Management Ltd) |
| 5889 | Salamol 100microgram/inhalation Inhalation powder (Kent Pharmaceuticals Ltd) |
| 5898 | Salamol steri-neb 2.5mg/2.5ml Nebuliser liquid (Numark Management Ltd) |
| 6081 | Ipratropium bromide 20micrograms/dose breath actuated inhaler |
| 6462 | Salbutamol 95micrograms/dose dry powder inhaler |
| 6512 | Atrovent 20micrograms/dose inhaler CFC free (Boehringer Ingelheim Ltd) |
| 6522 | Ipratropium bromide 20micrograms/dose inhaler CFC free |
| 6719 | Ipratropium bromide 500micrograms/2ml nebuliser liquid unit dose vials |
| 6758 | Ipratropium 250micrograms/1ml nebuliser liquid Steri-Neb unit dose vials (Teva UK Ltd) |
| 6772 | Ipratropium bromide 250micrograms/1ml nebuliser liquid unit dose vials |
| 6911 | Atrovent 250micrograms/1ml nebuliser liquid UDVs (Boehringer Ingelheim Ltd) |
| 7017 | Salbutamol 100micrograms/dose dry powder inhaler |
| 7140 | Atrovent 500micrograms/2ml nebuliser liquid UDVs (Boehringer Ingelheim Ltd) |
| 7711 | Terbutaline 250micrograms/dose inhaler with spacer |
| 7935 | Maxivent 100microgram/inhalation Inhalation powder (Ashbourne Pharmaceuticals Ltd) |
| 7953 | Terbutaline 1.5mg/5ml oral solution sugar free |
| 7954 | Bricanyl 250micrograms/dose spacer inhaler (AstraZeneca UK Ltd) |
| 7965 | Salbutamol 5mg/ml nebuliser liquid |
| 8267 | Sodium cromoglicate 1mg/dose / Salbutamol 100micrograms/dose inhaler |
| 8333 | Ipratropium bromide 40microgram inhalation powder capsules |
| 8522 | Terbutaline 7.5mg modified-release tablets |
| 8676 | Terbutaline 10mg/ml nebuliser liquid |
| 9270 | Ipratropium bromide with fenoterol hydrobromide 500micrograms + 1.25mg/4ml |
| 9384 | Salbutamol 4mg modified-release capsules |
| 9651 | Asmasal 100microgram/inhalation Spacehaler (Celltech Pharma Europe Ltd) |
| 9681 | Atrovent aerohaler 40microgram Inhalation powder (Boehringer Ingelheim Ltd) |
| 9805 | Salbutamol 5mg/50ml solution for infusion vials |
| 10360 | Aerocrom inhaler (Castlemead Healthcare Ltd) |
| 10458 | Ventolin cr 4mg Tablet (Allen & Hanburys Ltd) |
| 10825 | Terbutaline 5mg tablets |
| 11046 | Ipratropium bromide with salbutamol 500micrograms + 2.5mg/2.5ml |
| 11307 | Salbutamol 100micrograms/dose / Beclometasone 50micrograms/dose inhaler |
| 11779 | Ipratropium bromide 40microgram inhalation powder capsules with device |
| 12042 | Ventolin cr 8mg Tablet (Allen & Hanburys Ltd) |
| 12808 | Fenoterol 100micrograms/dose / Ipratropium bromide 40micrograms/dose breath actuated inhaler |
| 12822 | Salbutamol 2.5mg with ipratropium bromide 500micrograms/2.5ml unit dose nebuilser solution |
| 12909 | Salbutamol 100micrograms/dose / Ipratropium 20micrograms/dose inhaler |
| 12909 | Salbutamol 100micrograms/dose / Ipratropium 20micrograms/dose inhaler |
| 13038 | Pulvinal Salbutamol 200micrograms/dose dry powder inhaler (Chiesi Ltd) |
| 13181 | Easyhaler Salbutamol sulfate 100micrograms/dose dry powder inhaler (Orion Pharma (UK) Ltd) |
| 13307 | Bricanyl 500microgram/ml Injection (AstraZeneca UK Ltd) |
| 13757 | Tropiovent steripoule 250microgram/ml Nebuliser liquid (Ashbourne Pharmaceuticals Ltd) |
| 13996 | Salamol 100microgram/inhalation Inhalation powder (Sandoz Ltd) |
| 14483 | Terbutaline 500micrograms/ml injection |
| 14525 | Salbutamol 100micrograms/inhalation vortex inhaler |
| 14561 | Salbutamol 400microgram / Beclometasone 200microgram inhalation powder capsules |
| 15483 | Bricanyl Oral solution (AstraZeneca UK Ltd) |
| 15613 | Salbutamol 500micrograms/1ml solution for injection ampoules |
| 16207 | Duovent UDVs nebuliser liquid 4ml (Boehringer Ingelheim Ltd) |
| 16577 | Easyhaler Salbutamol sulfate 200micrograms/dose dry powder inhaler (Orion Pharma (UK) Ltd) |
| 16625 | Ventide Rotacaps (GlaxoSmithKline UK Ltd) |
| 17185 | Ventolin 500micrograms/1ml solution for injection ampoules (GlaxoSmithKline UK Ltd) |
| 17696 | Ventmax SR 4mg capsules (Chiesi Ltd) |
| 17874 | Monovent 1.5mg/5ml Oral solution (Lagap) |
| 17875 | Terbutaline with guafenesin expectorant |
| 18140 | Respontin 500micrograms/2ml Nebules (GlaxoSmithKline UK Ltd) |
| 18299 | Fenoterol 1.25mg/4ml / Ipratropium 500micrograms/4ml nebuliser liquid unit dose vials |
| 18314 | Aerocrom Syncroner with spacer (Castlemead Healthcare Ltd) |
| 18421 | Respontin nebules 250microgram/ml Nebuliser liquid (Glaxo Wellcome UK Ltd) |
| 18456 | Salbutamol 200microgram / Beclometasone 100microgram inhalation powder capsules |
| 18484 | Ventide Paediatric Rotacaps (GlaxoSmithKline UK Ltd) |
| 18622 | Salbulin 2mg Tablet (3M Health Care Ltd) |
| 18968 | Salbutamol 5mg/5ml solution for infusion ampoules |
| 19121 | Beclometasone 100micrograms with Salbutamol 200micrograms inhalation capsules |
| 19376 | Beclometasone 200micrograms with Salbutamol 400micrograms inhalation capsules |
| 20838 | Salbuvent 2mg Tablet (Pharmacia Ltd) |
| 21102 | Salbutamol 2mg/5ml Oral solution (Lagap) |
| 21859 | Asmaven 100microgram Inhalation powder (Berk Pharmaceuticals Ltd) |
| 22313 | Ventmax SR 8mg capsules (Chiesi Ltd) |
| 22430 | Spacehaler salbutamol 100microgram/inhalation Spacehaler (Celltech Pharma Europe Ltd) |
| 23269 | Maxivent 2.5mg/2.5ml nebuliser liquid unit dose Steripoule vials (Ashbourne Pharmaceuticals Ltd) |
| 23567 | Respontin 250micrograms/1ml Nebules (GlaxoSmithKline UK Ltd) |
| 23709 | Ipratropium 500micrograms/2ml nebuliser liquid Steri-Neb unit dose vials (Teva UK Ltd) |
| 23961 | Ipratropium bromide 250microgram/ml Inhalation vapour (Galen Ltd) |
| 24380 | Sodium cromoglicate 1mg/dose / Salbutamol 100micrograms/dose inhaler with spacer |
| 24645 | Ventolin 5mg/5ml solution for infusion ampoules (GlaxoSmithKline UK Ltd) |
| 25339 | Maxivent 5mg/2.5ml nebuliser liquid unit dose Steripoule vials (Ashbourne Pharmaceuticals Ltd) |
| 26616 | Ipratropium bromide with fenoterol hydrobromide 0micrograms + 100micrograms/actuation |
| 26873 | Cobutolin 2mg Tablet (Actavis UK Ltd) |
| 26987 | Bricanyl Tablet (AstraZeneca UK Ltd) |
| 27340 | Salbuvent 0.5mg/ml Injection (Pharmacia Ltd) |
| 27505 | Ipratropium bromide with fenoterol hydrobromide 40micrograms + 100micrograms/actuation |
| 28508 | Salbutamol 100microgram/inhalation Inhalation powder (IVAX Pharmaceuticals UK Ltd) |
| 28577 | Ventolin 50microgram/ml Injection (Allen & Hanburys Ltd) |
| 28881 | Salbutamol 2mg/5ml oral solution sugar free (A A H Pharmaceuticals Ltd) |
| 29267 | Salbuvent 4mg Tablet (Pharmacia Ltd) |
| 30118 | Salbutamol 100micrograms/dose inhaler CFC free (Teva UK Ltd) |
| 30204 | Salbutamol 200micrograms inahalation capsules |
| 30212 | Salbutamol cyclohaler |
| 30229 | Ipratropium bromide 250microgram/ml Nebuliser liquid (Galen Ltd) |
| 30230 | Salbutamol 100micrograms/actuation breath actuated inhaler |
| 30240 | Aerolin autohaler 100microgram/actuation Pressurised inhalation (3M Health Care Ltd) |
| 31082 | Salbuvent 5mg/ml Respirator solution (Pharmacia Ltd) |
| 31845 | Salapin 2mg/5ml syrup (Pinewood Healthcare) |
| 31933 | Salbutamol 100micrograms/dose inhaler (A A H Pharmaceuticals Ltd) |
| 32050 | Salbutamol 400 Cyclocaps (Teva UK Ltd) |
| 32102 | Salbutamol 4mg tablets (A A H Pharmaceuticals Ltd) |
| 33089 | Salbutamol 100micrograms/dose inhaler (Kent Pharmaceuticals Ltd) |
| 33373 | Salbutamol 200 Cyclocaps (Teva UK Ltd) |
| 33588 | Salbutamol 100micrograms/dose inhaler (Mylan Ltd) |
| 33817 | Salbutamol 100micrograms/dose inhaler CFC free (Actavis UK Ltd) |
| 34018 | Salbutamol 5mg/2.5ml Nebuliser liquid (Galen Ltd) |
| 34029 | Salbutamol 400micrograms inahalation capsules |
| 34134 | Aerolin 400 100microgram/actuation Inhalation powder (3M Health Care Ltd) |
| 34162 | Salbutamol 2.5mg/2.5ml Nebuliser liquid (Galen Ltd) |
| 34310 | Salbutamol 100micrograms/dose inhaler CFC free (A A H Pharmaceuticals Ltd) |
| 34311 | Salbutamol 100microgram/inhalation Inhalation powder (Berk Pharmaceuticals Ltd) |
| 34618 | Salbutamol 2mg tablets (Actavis UK Ltd) |
| 34619 | Salbutamol 100microgram/inhalation Inhalation powder (Kent Pharmaceuticals Ltd) |
| 34702 | Salbutamol 100microgram/inhalation Inhalation powder (C P Pharmaceuticals Ltd) |
| 34938 | Salbutamol 4mg tablets (Actavis UK Ltd) |
| 35522 | Bricanyl 500micrograms/1ml solution for injection ampoules (AstraZeneca UK Ltd) |
| 35557 | Ipramol nebuliser solution 2.5ml Steri-Neb unit dose vials (Teva UK Ltd) |
| 35744 | Bricanyl 2.5mg/5ml solution for injection ampoules (AstraZeneca UK Ltd) |
| 35861 | Terbutaline 2.5mg/5ml solution for injection ampoules |
| 35862 | Terbutaline 500micrograms/1ml solution for injection ampoules |
| 37612 | Terbutaline 5mg/2ml nebuliser liquid unit dose vials (Galen Ltd) |
| 37791 | Ipratropium bromide 250microgram/ml |
| 38079 | Salbutamol 100micrograms/dose dry powder inhalation cartridge with device |
| 38097 | Salbutamol cyclocaps 200microgram Inhalation powder (DuPont Pharmaceuticals Ltd) |
| 38136 | Salbulin Novolizer 100micrograms/dose inhalation powder (Meda Pharmaceuticals Ltd) |
| 38214 | Salbutamol 100micrograms/dose dry powder inhalation cartridge |
| 38226 | Salbulin Novolizer 100micrograms/dose inhalation powder refill (Meda Pharmaceuticals Ltd) |
| 38416 | Salbutamol cyclocaps 400microgram Inhalation powder (DuPont Pharmaceuticals Ltd) |
| 38419 | Terbutaline 1.5mg/5ml oral solution sugar free (A A H Pharmaceuticals Ltd) |
| 40177 | Ipratropium bromide 250microgram/ml Nebuliser liquid (Hillcross Pharmaceuticals Ltd) |
| 40599 | Salbutamol 5mg/2.5ml nebuliser liquid unit dose Steripoule vials (Galen Ltd) |
| 40637 | Ipratropium 250micrograms/1ml nebuliser liquid unit dose Steripoule vials (Galen Ltd) |
| 40655 | Salbuvent 100microgram/actuation Inhalation powder (Pharmacia Ltd) |
| 40709 | Salbutamol 2.5mg/2.5ml nebuliser liquid unit dose vials (A A H Pharmaceuticals Ltd) |
| 40832 | Ipratropium 500micrograms/2ml nebuliser liquid unit dose Steripoule vials (Galen Ltd) |
| 41548 | Salbutamol 2mg tablets (Approved Prescription Services Ltd) |
| 41549 | Salbutamol 2mg Tablet (C P Pharmaceuticals Ltd) |
| 41691 | Salbutamol 2mg/5ml oral solution sugar free (Sandoz Ltd) |
| 41832 | Monovent 1.5mg/5ml syrup (Sandoz Ltd) |
| 42279 | Salbutamol 2.5mg/2.5ml nebuliser liquid unit dose Steripoule vials (Galen Ltd) |
| 42497 | Salbutamol 8mg tablet |
| 42830 | Ventolin 100micrograms/dose Evohaler (GlaxoSmithKline UK Ltd) |
| 42858 | Ventolin 200micrograms/dose Accuhaler (GlaxoSmithKline UK Ltd) |
| 42867 | Terbutaline 1.5mg/5ml Oral solution (Sandoz Ltd) |
| 42886 | Bricanyl 500micrograms/dose Turbohaler (AstraZeneca UK Ltd) |
| 43046 | Salipraneb 0.5mg/2.5mg nebuliser solution 2.5ml ampoules (Arrow Generics Ltd) |
| 43085 | Bricanyl 5mg/2ml Respules (AstraZeneca UK Ltd) |
| 43090 | Atrovent 40microgram Aerocaps (Boehringer Ingelheim Ltd) |
| 43105 | Atrovent 40microgram Aerocaps with Aerohaler (Boehringer Ingelheim Ltd) |
| 44713 | Salbutamol 100microgram/inhalation Inhalation powder (Celltech Pharma Europe Ltd) |
| 45863 | Salbutamol 5mg/2.5ml Nebuliser liquid (Generics (UK) Ltd) |
| 46551 | Salbutamol 100microgram/inhalation Inhalation powder (Neo Laboratories Ltd) |
| 48410 | Salbutamol 2.5mg/2.5ml / Ipratropium bromide 500micrograms/2.5ml nebuliser liquid ampoules |
| 48490 | Ventolin 100micrograms/dose Evohaler (DE Pharmaceuticals) |
| 48519 | Ventolin 100micrograms/dose Evohaler (Waymade Healthcare Plc) |
| 48547 | Salamol 100micrograms/dose inhaler CFC free (Arrow Generics Ltd) |
| 48607 | Salbutamol 2.5mg/2.5ml / Ipratropium bromide 500micrograms/2.5ml nebuliser liquid unit dose vials |
| 48741 | Ventolin 100micrograms/dose Evohaler (Mawdsley-Brooks & Company Ltd) |
| 48742 | Ventodisks 400microgram (GlaxoSmithKline UK Ltd) |
| 48809 | Ventodisks 400microgram with Diskhaler (GlaxoSmithKline UK Ltd) |
| 49368 | Ventodisks 200microgram with Diskhaler (GlaxoSmithKline UK Ltd) |
| 49369 | Salbutamol 200microgram inhalation powder blisters |
| 49370 | Ventodisks 200microgram (GlaxoSmithKline UK Ltd) |
| 49591 | Salbutamol 100micrograms/dose inhaler CFC free (Sandoz Ltd) |
| 49904 | Combivent nebuliser liquid 2.5ml UDVs (Lexon (UK) Ltd) |
| 50315 | Salbutamol 200microgram inhalation powder blisters with device |
| 50503 | Ventolin 200micrograms/dose Accuhaler (Mawdsley-Brooks & Company Ltd) |
| 50557 | Ventolin 200micrograms/dose Accuhaler (Lexon (UK) Ltd) |
| 50810 | Atrovent 20micrograms/dose inhaler CFC free (DE Pharmaceuticals) |
| 50956 | Ventolin 200micrograms/dose Accuhaler (DE Pharmaceuticals) |
| 51903 | Combivent nebuliser liquid 2.5ml UDVs (DE Pharmaceuticals) |
| 52410 | Bricanyl 500micrograms/dose Turbohaler (Necessity Supplies Ltd) |
| 52543 | Salbutamol 400microgram inhalation powder blisters |
| 52799 | Salbutamol 400microgram inhalation powder blisters with device |
| 53019 | Ventolin 2.5mg Nebules (Mawdsley-Brooks & Company Ltd) |
| 53174 | Ipratropium bromide 500micrograms/2ml nebuliser liquid unit dose vials (A A H Pharmaceuticals Ltd) |
| 53297 | Ventolin 200micrograms/dose Accuhaler (Sigma Pharmaceuticals Plc) |
| 55132 | Atrovent 500micrograms/2ml nebuliser liquid UDVs (Waymade Healthcare Plc) |
| 57249 | Asmavent 100micrograms/dose inhaler CFC free (Kent Pharmaceuticals Ltd) |
| 57524 | Ventolin 200micrograms/dose Accuhaler (Dowelhurst Ltd) |
| 57557 | Atrovent 20micrograms/dose inhaler CFC free (Lexon (UK) Ltd) |
| 58269 | AirSalb 100micrograms/dose inhaler CFC free (Sandoz Ltd) |
| 59409 | Salbutamol 100micrograms/dose inhaler CFC free (Waymade Healthcare Plc) |
| 60601 | Salbutamol 5mg/2.5ml nebuliser liquid unit dose vials (Alliance Healthcare (Distribution) Ltd) |
| 60920 | Atrovent 20micrograms/dose inhaler CFC free (Sigma Pharmaceuticals Plc) |
| 60923 | Salamol 100micrograms/dose Easi-Breathe inhaler (DE Pharmaceuticals) |
| 61330 | Salbutamol 2.5mg/2.5ml nebuliser liquid unit dose vials (Alliance Healthcare (Distribution) Ltd) |
| 61591 | Salbutamol 100micrograms/dose inhaler CFC free (Phoenix Healthcare Distribution Ltd) |
| 64801 | Salbutamol 100micrograms/dose inhaler CFC free (Mylan Ltd) |

Table S4: COPD medical codes

| **Medcode** | **Read Code** | **Read Term** |
| --- | --- | --- |
| 11287 | 66YM.00 | Chronic obstructive pulmonary disease annual review |
| 1001 | H3...00 | Chronic obstructive pulmonary disease |
| 998 | H3...11 | Chronic obstructive airways disease |
| 10863 | H36..00 | Mild chronic obstructive pulmonary disease |
| 5710 | H3z..00 | Chronic obstructive airways disease NOS |
| 10802 | H37..00 | Moderate chronic obstructive pulmonary disease |
| 794 | H32..00 | Emphysema |
| 9876 | H38..00 | Severe chronic obstructive pulmonary disease |
| 21061 | H3y0.00 | Chronic obstruct pulmonary dis with acute lower resp infectn |
| 93568 | H39..00 | Very severe chronic obstructive pulmonary disease |
| 14798 | H312100 | Emphysematous bronchitis |
| 12166 | H3y..00 | Other specified chronic obstructive airways disease |
| 27819 | H312.00 | Obstructive chronic bronchitis |
| 33450 | H32z.00 | Emphysema NOS |
| 45771 | 66Yh.00 | Chronic obstructive pulmonary disease does not disturb sleep |
| 37247 | H3z..11 | Chronic obstructive pulmonary disease NOS |
| 26306 | H320.00 | Chronic bullous emphysema |
| 45770 | 66Yg.00 | Chronic obstructive pulmonary disease disturbs sleep |
| 15782 | G41z.00 | Chronic pulmonary heart disease NOS |
| 23492 | H320z00 | Chronic bullous emphysema NOS |
| 44525 | H312z00 | Obstructive chronic bronchitis NOS |
| 16410 | H32yz00 | Other emphysema NOS |
| 65733 | Hyu3100 | [X]Other specified chronic obstructive pulmonary disease |
| 40788 | H32y.00 | Other emphysema |
| 26082 | H541000 | Chronic pulmonary oedema |
| 46578 | H321.00 | Panlobular emphysema |
| 60188 | H320200 | Giant bullous emphysema |
| 56860 | H320000 | Segmental bullous emphysema |
| 68662 | H320100 | Zonal bullous emphysema |
| 54893 | H582.00 | Compensatory emphysema |
| 99536 | H320300 | Bullous emphysema with collapse |
| 66058 | Hyu3000 | [X]Other emphysema |
| 67040 | H3y..11 | Other specified chronic obstructive pulmonary disease |
| 64721 | H464000 | Chronic emphysema due to chemical fumes |
| 70787 | H32y100 | Atrophic (senile) emphysema |
| 10863 | H36..00 | Mild chronic obstructive pulmonary disease |
| 10802 | H37..00 | Moderate chronic obstructive pulmonary disease |
| 9876 | H38..00 | Severe chronic obstructive pulmonary disease |
| 93568 | H39..00 | Very severe chronic obstructive pulmonary disease |
| 3243 | H31..00 | Chronic bronchitis |
| 15157 | H31x.00 | Chronic bronchitis NOS |
| 25603 | H310.00 | Simple chronic bronchitis |
| 61118 | H310z00 | Simple chronic bronchitis NOS |
| 66043 | H31y.00 | Other chronic bronchitis |
| 68066 | H31yz00 | Other chronic bronchitis NOS |

Table S5: Co-morbidities codes

| **Medcode** | **Read Code** | **Read Term** |
| --- | --- | --- |
| **Asthma** | | |
| 78 | H33..00 | Asthma |
| 185 | H333.00 | Acute exacerbation of asthma |
| 13064 | 663V.00 | Asthma severity |
| 16070 | H33zz00 | Asthma NOS |
| 232 | H33z100 | Asthma attack |
| 10318 | 1J70.00 | Suspected asthma |
| 1555 | H33..11 | Bronchial asthma |
| 4442 | H33z.00 | Asthma unspecified |
| 233 | H33z011 | Severe asthma attack |
| 7058 | 8H2P.00 | Emergency admission asthma |
| 9018 | 663y.00 | Number of asthma exacerbations in past year |
| 7146 | H330.00 | Extrinsic (atopic) asthma |
| 1208 | H330.12 | Childhood asthma |
| 3018 | 663V100 | Mild asthma |
| 13066 | 663h.00 | Asthma - currently dormant |
| 2290 | H330.11 | Allergic asthma |
| 13065 | 663V200 | Moderate asthma |
| 10487 | 663j.00 | Asthma - currently active |
| 3458 | 663V000 | Occasional asthma |
| 11370 | 1O2..00 | Asthma confirmed |
| 5267 | H331.00 | Intrinsic asthma |
| 3665 | H331.11 | Late onset asthma |
| 4892 | H33z000 | Status asthmaticus NOS |
| 6707 | H330111 | Extrinsic asthma with asthma attack |
| 12987 | H33z200 | Late-onset asthma |
| 14777 | H330000 | Extrinsic asthma without status asthmaticus |
| 3366 | 663V300 | Severe asthma |
| 8335 | H33z111 | Asthma attack NOS |
| 5798 | H312000 | Chronic asthmatic bronchitis |
| 45782 | H330z00 | Extrinsic asthma NOS |
| 25796 | H332.00 | Mixed asthma |
| 40823 | H334.00 | Brittle asthma |
| 22752 | 173c.00 | Occupational asthma |
| 23481 | G581.11 | Asthma - cardiac |
| 29325 | H331000 | Intrinsic asthma without status asthmaticus |
| 45073 | H331z00 | Intrinsic asthma NOS |
| 27926 | H330100 | Extrinsic asthma with status asthmaticus |
| 18323 | H331111 | Intrinsic asthma with asthma attack |
| 21232 | H33zz12 | Allergic asthma NEC |
| 58196 | H331100 | Intrinsic asthma with status asthmaticus |
| 73522 | 173d.00 | Work aggravated asthma |
| 39478 | H35y700 | Wood asthma |
| 40864 | U60F615 | [X] Adverse reaction to theophylline - asthma |
| 93353 | H35y600 | Sequoiosis (red-cedar asthma) |
| 26501 | 663s.00 | Asthma never causes daytime symptoms |
| 26503 | 663v.00 | Asthma causes daytime symptoms most days |
| 31225 | 663t.00 | Asthma causes daytime symptoms 1 to 2 times per month |
| 24884 | 663u.00 | Asthma causes daytime symptoms 1 to 2 times per week |
| 42824 | 663q.00 | Asthma daytime symptoms |
| 31167 | 66YP.00 | Asthma night-time symptoms |
| 30815 | 663N000 | Asthma causing night waking |
| 39570 | 663r.00 | Asthma causes night symptoms 1 to 2 times per month |
| 102400 | 66Yq.00 | Asthma causes night time symptoms 1 to 2 times per week |
| 102395 | 66Yr.00 | Asthma causes symptoms most nights |
| 103612 | 66Ys.00 | Asthma never causes night symptoms |
| 13173 | 663O.00 | Asthma not disturbing sleep |
| 13174 | 663Q.00 | Asthma not limiting activities |
| 26504 | 663f.00 | Asthma never restricts exercise |
| 38143 | 663O000 | Asthma never disturbs sleep |
| 26861 | 663e000 | Asthma sometimes restricts exercise |
| 7416 | 663N.00 | Asthma disturbing sleep |
| 7191 | 663P.00 | Asthma limiting activities |
| 38144 | 663w.00 | Asthma limits walking up hills or stairs |
| 25181 | 663e.00 | Asthma restricts exercise |
| 13175 | 663N200 | Asthma disturbs sleep frequently |
| 38146 | 663N100 | Asthma disturbs sleep weekly |
| 26506 | 663e100 | Asthma severely restricts exercise |
| 38145 | 663x.00 | Asthma limits walking on the flat |
| 103998 | 663P200 | Asthma limits activities most days |
| 102713 | 663P000 | Asthma limits activities 1 to 2 times per month |
| 102888 | 663P100 | Asthma limits activities 1 to 2 times per week |
| 13176 | 66YK.00 | Asthma follow-up |
| 719 | 14B4.00 | H/O: asthma |
| 19520 | 663n.00 | Asthma treatment compliance satisfactory |
| 41020 | 66YC.00 | Absent from work or school due to asthma |
| 100509 | 9NNX.00 | Under care of asthma specialist nurse |
| 5627 | H330011 | Hay fever with asthma |
| 15248 | H330.13 | Hay fever with asthma |
| 11022 | 178..00 | Asthma trigger |
| 5867 | 173A.00 | Exercise induced asthma |
| 102449 | 1789.00 | Asthma trigger - respiratory infection |
| 102341 | 1781.00 | Asthma trigger - pollen |
| 4606 | H33zz11 | Exercise induced asthma |
| 103813 | 1788.00 | Asthma trigger - cold air |
| 102871 | 178B.00 | Asthma trigger - exercise |
| 103944 | 178A.00 | Asthma trigger - airborne dust |
| 103321 | 1786.00 | Asthma trigger - animals |
| 102301 | 1787.00 | Asthma trigger - seasonal |
| 7731 | H330.14 | Pollen asthma |
| 103945 | 1785.00 | Asthma trigger - damp |
| 102952 | 1783.00 | Asthma trigger - warm air |
| 103952 | 1784.00 | Asthma trigger - emotion |
| 103955 | 1782.00 | Asthma trigger - tobacco smoke |
| 41017 | 1780.00 | Aspirin induced asthma |
| 47684 | H47y000 | Detergent asthma |
| 47337 | 663m.00 | Asthma accident and emergency attendance since last visit |
| 24479 | 663d.00 | Emergency asthma admission since last appointment |
| 16667 | 8795 | Asthma control step 2 |
| 18224 | 8796 | Asthma control step 3 |
| 16785 | 8794 | Asthma control step 1 |
| 29645 | 8793 | Asthma control step 0 |
| 98185 | 38DL.00 | Asthma control test |
| 20886 | 8797 | Asthma control step 4 |
| 20860 | 8798 | Asthma control step 5 |
| 100397 | 38DT.00 | Asthma control questionnaire |
| **Stroke** | | |
| 1469 | G66..00 | Stroke and cerebrovascular accident unspecified |
| 1298 | G66..11 | CVA unspecified |
| 10792 | 662M.00 | Stroke monitoring |
| 11039 | 9h21.00 | Excepted from stroke quality indicators: Patient unsuitable |
| 18686 | 662e.00 | Stroke/CVA annual review |
| 28753 | 9Om0.00 | Stroke/transient ischaemic attack monitoring first letter |
| 3149 | G64z.00 | Cerebral infarction NOS |
| 5363 | G64..11 | CVA - cerebral artery occlusion |
| 6116 | G66..13 | CVA - Cerebrovascular accident unspecified |
| 6305 | 14A7.11 | H/O: CVA |
| 6960 | G61..11 | CVA - cerebrovascular accid due to intracerebral haemorrhage |
| 5871 | 14A7.12 | H/O: stroke |
| 34245 | 9Om1.00 | Stroke/transient ischaemic attack monitoring second letter |
| 34135 | 14A7.00 | H/O: CVA/stroke |
| 569 | G64..12 | Infarction - cerebral |
| 31218 | 9Om..00 | Stroke/transient ischaemic attack monitoring administration |
| 7780 | G667.00 | Left sided CVA |
| 34375 | 9Om2.00 | Stroke/transient ischaemic attack monitoring third letter |
| 12833 | G668.00 | Right sided CVA |
| 6155 | G64..13 | Stroke due to cerebral arterial occlusion |
| 6253 | G66..12 | Stroke unspecified |
| 18604 | G61..12 | Stroke due to intracerebral haemorrhage |
| 17322 | G664.00 | Cerebellar stroke syndrome |
| 8443 | G663.00 | Brain stem stroke syndrome |
| 9985 | G64z200 | Left sided cerebral infarction |
| 10504 | G64z300 | Right sided cerebral infarction |
| 36717 | G640000 | Cerebral infarction due to thrombosis of cerebral arteries |
| 23671 | G63y000 | Cerebral infarct due to thrombosis of precerebral arteries |
| 7138 | ZV12512 | [V]Personal history of cerebrovascular accident (CVA) |
| 19348 | ZV12511 | [V]Personal history of stroke |
| 6228 | G68X.00 | Sequelae of stroke,not specfd as h'morrhage or infarction |
| 24446 | G63y100 | Cerebral infarction due to embolism of precerebral arteries |
| 89913 | 9Om4.00 | Stroke/transient ischaemic attack monitoring telephone invte |
| 39344 | G676000 | Cereb infarct due cerebral venous thrombosis, nonpyogenic |
| 27975 | G641000 | Cerebral infarction due to embolism of cerebral arteries |
| 53745 | Gyu6400 | [X]Other cerebral infarction |
| 39403 | G683.00 | Sequelae of cerebral infarction |
| 28914 | 662o.00 | Haemorrhagic stroke monitoring |
| 47607 | L440.11 | CVA - cerebrovascular accident in the puerperium |
| 66873 | 14AK.00 | H/O: Stroke in last year |
| 51759 | G677000 | Occlusion and stenosis of middle cerebral artery |
| 56458 | 8HHM.00 | Ref to multidisciplinary stroke function improvement service |
| 31704 | G677.00 | Occlusion/stenosis cerebral arts not result cerebral infarct |
| 55351 | 7P24200 | Delivery of rehabilitation for stroke |
| 65770 | G677200 | Occlusion and stenosis of posterior cerebral artery |
| 57527 | G677100 | Occlusion and stenosis of anterior cerebral artery |
| 56279 | L440.12 | Stroke in the puerperium |
| **Myocardial infarction** | | |
| 241 | G30..00 | Acute myocardial infarction |
| 1677 | G30..15 | MI - acute myocardial infarction |
| 14658 | G30z.00 | Acute myocardial infarction NOS |
| 10562 | G307100 | Acute non-ST segment elevation myocardial infarction |
| 12229 | G30X000 | Acute ST segment elevation myocardial infarction |
| 1678 | G308.00 | Inferior myocardial infarction NOS |
| 1204 | G30..14 | Heart attack |
| 2491 | G30..12 | Coronary thrombosis |
| 3704 | G307.00 | Acute subendocardial infarction |
| 14897 | G301z00 | Anterior myocardial infarction NOS |
| 5387 | G301.00 | Other specified anterior myocardial infarction |
| 12139 | G300.00 | Acute anterolateral infarction |
| 8935 | G302.00 | Acute inferolateral infarction |
| 17872 | G301100 | Acute anteroseptal infarction |
| 9507 | G307000 | Acute non-Q wave infarction |
| 23892 | G304.00 | Posterior myocardial infarction NOS |
| 14898 | G305.00 | Lateral myocardial infarction NOS |
| 29643 | G303.00 | Acute inferoposterior infarction |
| 13566 | G30..11 | Attack - heart |
| 17689 | G30..17 | Silent myocardial infarction |
| 13571 | G30..16 | Thrombosis - coronary |
| 46017 | G30yz00 | Other acute myocardial infarction NOS |
| 18842 | G35..00 | Subsequent myocardial infarction |
| 32272 | G38..00 | Postoperative myocardial infarction |
| 34803 | G30y.00 | Other acute myocardial infarction |
| 41221 | G30y200 | Acute septal infarction |
| 30421 | G30..13 | Cardiac rupture following myocardial infarction (MI) |
| 29758 | G30X.00 | Acute transmural myocardial infarction of unspecif site |
| 40429 | G301000 | Acute anteroapical infarction |
| 30330 | G309.00 | Acute Q-wave infarct |
| 28736 | G30y000 | Acute atrial infarction |
| 45809 | G350.00 | Subsequent myocardial infarction of anterior wall |
| 32854 | G30B.00 | Acute posterolateral myocardial infarction |
| 63467 | G306.00 | True posterior myocardial infarction |
| 38609 | G351.00 | Subsequent myocardial infarction of inferior wall |
| 36423 | G36..00 | Certain current complication follow acute myocardial infarct |
| 41835 | G384.00 | Postoperative subendocardial myocardial infarction |
| 46276 | G381.00 | Postoperative transmural myocardial infarction inferior wall |
| 61670 | 889A.00 | Diab mellit insulin-glucose infus acute myocardial infarct |
| 68748 | G38z.00 | Postoperative myocardial infarction, unspecified |
| 46166 | G35X.00 | Subsequent myocardial infarction of unspecified site |
| 72562 | G353.00 | Subsequent myocardial infarction of other sites |
| 46112 | G380.00 | Postoperative transmural myocardial infarction anterior wall |
| 69474 | G365.00 | Rupture papillary muscle/curr comp fol acute myocard infarct |
| 99991 | Gyu3600 | [X]Subsequent myocardial infarction of unspecified site |
| 62626 | G30y100 | Acute papillary muscle infarction |
| 59940 | G364.00 | Ruptur chordae tendinae/curr comp fol acute myocard infarct |
| 96838 | Gyu3400 | [X]Acute transmural myocardial infarction of unspecif site |
| 106812 | G383.00 | Postoperative transmural myocardial infarction unspec site |
| **Diabetes** | | |
| 1549 | C10E.00 | Type 1 diabetes mellitus |
| 8842 | 66A5.00 | Diabetic on insulin |
| 1038 | C100011 | Insulin dependent diabetes mellitus |
| 1647 | C108.00 | Insulin dependent diabetes mellitus |
| 17858 | C108.12 | Type 1 diabetes mellitus |
| 10692 | C10EM00 | Type 1 diabetes mellitus with ketoacidosis |
| 24423 | C108.13 | Type I diabetes mellitus |
| 24490 | C100000 | Diabetes mellitus, juvenile type, no mention of complication |
| 10418 | C10ED00 | Type 1 diabetes mellitus with nephropathy |
| 16946 | 13L4.11 | Diabetic child |
| 30323 | C10EK00 | Type 1 diabetes mellitus with persistent proteinuria |
| 30294 | C10EL00 | Type 1 diabetes mellitus with persistent microalbuminuria |
| 50960 | L180500 | Pre-existing diabetes mellitus, insulin-dependent |
| 18387 | C10E700 | Type 1 diabetes mellitus with retinopathy |
| 6509 | C108700 | Insulin dependent diabetes mellitus with retinopathy |
| 44443 | C108500 | Insulin dependent diabetes mellitus with ulcer |
| 6791 | C108800 | Insulin dependant diabetes mellitus - poor control |
| 51261 | C10E.12 | Insulin dependent diabetes mellitus |
| 32359 | ZRbH.00 | Perceived control of insulin-dependent diabetes |
| 35288 | C10E800 | Type 1 diabetes mellitus - poor control |
| 40837 | C10EN00 | Type 1 diabetes mellitus with ketoacidotic coma |
| 46624 | C10C.11 | Maturity onset diabetes in youth |
| 26855 | C108400 | Unstable insulin dependant diabetes mellitus |
| 53200 | C101000 | Diabetes mellitus, juvenile type, with ketoacidosis |
| 39070 | C10EE00 | Type 1 diabetes mellitus with hypoglycaemic coma |
| 12455 | C10E.11 | Type I diabetes mellitus |
| 55239 | C10EQ00 | Type 1 diabetes mellitus with gastroparesis |
| 44440 | C108E00 | Insulin dependent diabetes mellitus with hypoglycaemic coma |
| 22871 | C10EP00 | Type 1 diabetes mellitus with exudative maculopathy |
| 18683 | C10E500 | Type 1 diabetes mellitus with ulcer |
| 54008 | C10EJ00 | Type 1 diabetes mellitus with neuropathic arthropathy |
| 46963 | C108000 | Insulin-dependent diabetes mellitus with renal complications |
| 43921 | C10E400 | Unstable type 1 diabetes mellitus |
| 47582 | C10E000 | Type 1 diabetes mellitus with renal complications |
| 93380 | C10N100 | Cystic fibrosis related diabetes mellitus |
| 40682 | C10E900 | Type 1 diabetes mellitus maturity onset |
| 42729 | C108E11 | Type I diabetes mellitus with hypoglycaemic coma |
| 42831 | C10E200 | Type 1 diabetes mellitus with neurological complications |
| 46301 | C10EC00 | Type 1 diabetes mellitus with polyneuropathy |
| 47650 | C10E300 | Type 1 diabetes mellitus with multiple complications |
| 57621 | C108D00 | Insulin dependent diabetes mellitus with nephropathy |
| 38161 | C108711 | Type I diabetes mellitus with retinopathy |
| 41716 | C108C00 | Insulin dependent diabetes mellitus with polyneuropathy |
| 49276 | C108100 | Insulin-dependent diabetes mellitus with ophthalmic comps |
| 49554 | C10EF00 | Type 1 diabetes mellitus with diabetic cataract |
| 51957 | C108511 | Type I diabetes mellitus with ulcer |
| 41049 | C108712 | Type 1 diabetes mellitus with retinopathy |
| 67853 | C106000 | Diabetes mellitus, juvenile, + neurological manifestation |
| 44260 | C108F00 | Insulin dependent diabetes mellitus with diabetic cataract |
| 47649 | C10E100 | Type 1 diabetes mellitus with ophthalmic complications |
| 52283 | C108200 | Insulin-dependent diabetes mellitus with neurological comps |
| 56448 | C108A00 | Insulin-dependent diabetes without complication |
| 60499 | C108600 | Insulin dependent diabetes mellitus with gangrene |
| 62209 | C10EM11 | Type I diabetes mellitus with ketoacidosis |
| 69676 | C10EA00 | Type 1 diabetes mellitus without complication |
| 18642 | C10EH00 | Type 1 diabetes mellitus with arthropathy |
| 45276 | C10E312 | Insulin dependent diabetes mellitus with multiple complicat |
| 21983 | C108012 | Type 1 diabetes mellitus with renal complications |
| 40023 | C102000 | Diabetes mellitus, juvenile type, with hyperosmolar coma |
| 42567 | C103000 | Diabetes mellitus, juvenile type, with ketoacidotic coma |
| 69993 | C10E600 | Type 1 diabetes mellitus with gangrene |
| 49949 | C10E411 | Unstable type I diabetes mellitus |
| 68792 | C10z000 | Diabetes mellitus, juvenile type, + unspecified complication |
| 72345 | C102z00 | Diabetes mellitus NOS with hyperosmolar coma |
| 96235 | C10E911 | Type I diabetes mellitus maturity onset |
| 17545 | C108F11 | Type I diabetes mellitus with diabetic cataract |
| 18230 | C108J12 | Type 1 diabetes mellitus with neuropathic arthropathy |
| 52104 | C108300 | Insulin dependent diabetes mellitus with multiple complicatn |
| 54600 | C10E412 | Unstable insulin dependent diabetes mellitus |
| 60208 | C108J11 | Type I diabetes mellitus with neuropathic arthropathy |
| 61344 | C108011 | Type I diabetes mellitus with renal complications |
| 66872 | C108D11 | Type I diabetes mellitus with nephropathy |
| 69748 | C105000 | Diabetes mellitus, juvenile type, + ophthalmic manifestation |
| 45914 | C108812 | Type 1 diabetes mellitus - poor control |
| 49146 | C108211 | Type I diabetes mellitus with neurological complications |
| 60107 | C108411 | Unstable type I diabetes mellitus |
| 61829 | C108212 | Type 1 diabetes mellitus with neurological complications |
| 65616 | C108H00 | Insulin dependent diabetes mellitus with arthropathy |
| 68105 | C10EB00 | Type 1 diabetes mellitus with mononeuropathy |
| 68390 | C108512 | Type 1 diabetes mellitus with ulcer |
| 70448 | C107000 | Diabetes mellitus, juvenile +peripheral circulatory disorder |
| 70766 | C108E12 | Type 1 diabetes mellitus with hypoglycaemic coma |
| 93875 | C10E712 | Insulin dependent diabetes mellitus with retinopathy |
| 93878 | C10E511 | Type I diabetes mellitus with ulcer |
| 95343 | C10E711 | Type I diabetes mellitus with retinopathy |
| 24694 | C108B00 | Insulin dependent diabetes mellitus with mononeuropathy |
| 62352 | C108H11 | Type I diabetes mellitus with arthropathy |
| 62613 | C10EA11 | Type I diabetes mellitus without complication |
| 63017 | C108911 | Type I diabetes mellitus maturity onset |
| 66145 | C10EN11 | Type I diabetes mellitus with ketoacidotic coma |
| 72702 | C10E812 | Insulin dependent diabetes mellitus - poor control |
| 91942 | C10E311 | Type I diabetes mellitus with multiple complications |
| 91943 | C10EC11 | Type I diabetes mellitus with polyneuropathy |
| 93468 | C10EG00 | Type 1 diabetes mellitus with peripheral angiopathy |
| 93922 | C104000 | Diabetes mellitus, juvenile type, with renal manifestation |
| 95992 | C108A11 | Type I diabetes mellitus without complication |
| 758 | C10F.00 | Type 2 diabetes mellitus |
| 506 | C100112 | Non-insulin dependent diabetes mellitus |
| 4513 | C109.00 | Non-insulin dependent diabetes mellitus |
| 17859 | C109.12 | Type 2 diabetes mellitus |
| 1407 | C10FJ00 | Insulin treated Type 2 diabetes mellitus |
| 18219 | C109.13 | Type II diabetes mellitus |
| 18390 | C10FM00 | Type 2 diabetes mellitus with persistent microalbuminuria |
| 5884 | C109.11 | NIDDM - Non-insulin dependent diabetes mellitus |
| 18278 | C109J00 | Insulin treated Type 2 diabetes mellitus |
| 26054 | C10FL00 | Type 2 diabetes mellitus with persistent proteinuria |
| 12640 | C10FC00 | Type 2 diabetes mellitus with nephropathy |
| 22884 | C10F.11 | Type II diabetes mellitus |
| 18496 | C10F600 | Type 2 diabetes mellitus with retinopathy |
| 8403 | C109700 | Non-insulin dependant diabetes mellitus - poor control |
| 25627 | C10F700 | Type 2 diabetes mellitus - poor control |
| 32627 | C10FN00 | Type 2 diabetes mellitus with ketoacidosis |
| 34912 | C109400 | Non-insulin dependent diabetes mellitus with ulcer |
| 47954 | C10F900 | Type 2 diabetes mellitus without complication |
| 34450 | C10FK00 | Hyperosmolar non-ketotic state in type 2 diabetes mellitus |
| 36695 | C10D.00 | Diabetes mellitus autosomal dominant type 2 |
| 29979 | C109900 | Non-insulin-dependent diabetes mellitus without complication |
| 34268 | C10F200 | Type 2 diabetes mellitus with neurological complications |
| 53392 | C10F911 | Type II diabetes mellitus without complication |
| 18777 | C10F000 | Type 2 diabetes mellitus with renal complications |
| 35385 | C10FH00 | Type 2 diabetes mellitus with neuropathic arthropathy |
| 25591 | C10FQ00 | Type 2 diabetes mellitus with exudative maculopathy |
| 17262 | C109600 | Non-insulin-dependent diabetes mellitus with retinopathy |
| 18425 | C10FB00 | Type 2 diabetes mellitus with polyneuropathy |
| 60796 | C10FL11 | Type II diabetes mellitus with persistent proteinuria |
| 49074 | C10F400 | Type 2 diabetes mellitus with ulcer |
| 41389 | C105100 | Diabetes mellitus, adult onset, + ophthalmic manifestation |
| 63762 | C10z100 | Diabetes mellitus, adult onset, + unspecified complication |
| 44982 | C10FE00 | Type 2 diabetes mellitus with diabetic cataract |
| 50609 | L180600 | Pre-existing diabetes mellitus, non-insulin-dependent |
| 59365 | C109C00 | Non-insulin dependent diabetes mellitus with nephropathy |
| 46917 | C10FD00 | Type 2 diabetes mellitus with hypoglycaemic coma |
| 47321 | C10F100 | Type 2 diabetes mellitus with ophthalmic complications |
| 62674 | C10FA00 | Type 2 diabetes mellitus with mononeuropathy |
| 47315 | C10F711 | Type II diabetes mellitus - poor control |
| 18264 | C109J12 | Insulin treated Type II diabetes mellitus |
| 24458 | C109711 | Type II diabetes mellitus - poor control |
| 12736 | C10F500 | Type 2 diabetes mellitus with gangrene |
| 36633 | C109K00 | Hyperosmolar non-ketotic state in type 2 diabetes mellitus |
| 63690 | C10FR00 | Type 2 diabetes mellitus with gastroparesis |
| 64668 | C10FJ11 | Insulin treated Type II diabetes mellitus |
| 45913 | C109712 | Type 2 diabetes mellitus - poor control |
| 51756 | C10FP00 | Type 2 diabetes mellitus with ketoacidotic coma |
| 58604 | C109611 | Type II diabetes mellitus with retinopathy |
| 49655 | C10F611 | Type II diabetes mellitus with retinopathy |
| 37806 | C10FF00 | Type 2 diabetes mellitus with peripheral angiopathy |
| 59253 | C10FG00 | Type 2 diabetes mellitus with arthropathy |
| 37648 | C109J11 | Insulin treated non-insulin dependent diabetes mellitus |
| 55075 | C109411 | Type II diabetes mellitus with ulcer |
| 52303 | C109000 | Non-insulin-dependent diabetes mellitus with renal comps |
| 42762 | C109612 | Type 2 diabetes mellitus with retinopathy |
| 24836 | C109C12 | Type 2 diabetes mellitus with nephropathy |
| 45467 | C109B00 | Non-insulin dependent diabetes mellitus with polyneuropathy |
| 45919 | C109212 | Type 2 diabetes mellitus with neurological complications |
| 50429 | C109100 | Non-insulin-dependent diabetes mellitus with ophthalm comps |
| 69278 | C109E00 | Non-insulin depend diabetes mellitus with diabetic cataract |
| 40401 | C109500 | Non-insulin dependent diabetes mellitus with gangrene |
| 43785 | C109D00 | Non-insulin dependent diabetes mellitus with hypoglyca coma |
| 44779 | C109E12 | Type 2 diabetes mellitus with diabetic cataract |
| 47816 | C109H11 | Type II diabetes mellitus with neuropathic arthropathy |
| 55842 | C109200 | Non-insulin-dependent diabetes mellitus with neuro comps |
| 18209 | C109012 | Type 2 diabetes mellitus with renal complications |
| 48192 | C109E11 | Type II diabetes mellitus with diabetic cataract |
| 50225 | C109011 | Type II diabetes mellitus with renal complications |
| 62107 | C109511 | Type II diabetes mellitus with gangrene |
| 65267 | C10F300 | Type 2 diabetes mellitus with multiple complications |
| 65704 | C109412 | Type 2 diabetes mellitus with ulcer |
| 43227 | C10F311 | Type II diabetes mellitus with multiple complications |
| 46150 | C109512 | Type 2 diabetes mellitus with gangrene |
| 64571 | C109C11 | Type II diabetes mellitus with nephropathy |
| 66965 | C109H12 | Type 2 diabetes mellitus with neuropathic arthropathy |
| 24693 | C109G00 | Non-insulin dependent diabetes mellitus with arthropathy |
| 54899 | C109F11 | Type II diabetes mellitus with peripheral angiopathy |
| 61071 | C109D12 | Type 2 diabetes mellitus with hypoglycaemic coma |
| 67905 | C109211 | Type II diabetes mellitus with neurological complications |
| 47409 | C109B11 | Type II diabetes mellitus with polyneuropathy |
| 50527 | C10FB11 | Type II diabetes mellitus with polyneuropathy |
| 57278 | C10F011 | Type II diabetes mellitus with renal complications |
| 59725 | C109111 | Type II diabetes mellitus with ophthalmic complications |
| 60699 | C109F12 | Type 2 diabetes mellitus with peripheral angiopathy |
| 62146 | C109300 | Non-insulin-dependent diabetes mellitus with multiple comps |
| 72320 | C109A00 | Non-insulin dependent diabetes mellitus with mononeuropathy |
| 18143 | C109G11 | Type II diabetes mellitus with arthropathy |
| 49869 | C109G12 | Type 2 diabetes mellitus with arthropathy |
| 50813 | C109A11 | Type II diabetes mellitus with mononeuropathy |
| 56268 | C109D11 | Type II diabetes mellitus with hypoglycaemic coma |
| 70316 | C109112 | Type 2 diabetes mellitus with ophthalmic complications |
| 85991 | C10FM11 | Type II diabetes mellitus with persistent microalbuminuria |
| 91646 | C10F411 | Type II diabetes mellitus with ulcer |
| 93727 | C10FE11 | Type II diabetes mellitus with diabetic cataract |
| 711 | C10..00 | Diabetes mellitus |
| 7795 | C106.12 | Diabetes mellitus with neuropathy |
| 28769 | 66AV.00 | Diabetic on insulin and oral treatment |
| 16230 | C106.00 | Diabetes mellitus with neurological manifestation |
| 11626 | F420z00 | Diabetic retinopathy NOS |
| 16502 | C104.00 | Diabetes mellitus with renal manifestation |
| 11663 | M271100 | Neuropathic diabetic ulcer - foot |
| 31790 | F372.00 | Polyneuropathy in diabetes |
| 11551 | C10B.00 | Diabetes mellitus induced by steroids |
| 38986 | C100.00 | Diabetes mellitus with no mention of complication |
| 35399 | C107.00 | Diabetes mellitus with peripheral circulatory disorder |
| 27921 | 2G51000 | Foot abnormality - diabetes related |
| 15690 | C103.00 | Diabetes mellitus with ketoacidotic coma |
| 32403 | C107.11 | Diabetes mellitus with gangrene |
| 37315 | F3y0.00 | Diabetic mononeuropathy |
| 35785 | F372100 | Chronic painful diabetic neuropathy |
| 35107 | C104z00 | Diabetes mellitis with nephropathy NOS |
| 21482 | C102.00 | Diabetes mellitus with hyperosmolar coma |
| 34152 | G73y000 | Diabetic peripheral angiopathy |
| 2471 | K01x100 | Nephrotic syndrome in diabetes mellitus |
| 22573 | C106z00 | Diabetes mellitus NOS with neurological manifestation |
| 5002 | F372.11 | Diabetic polyneuropathy |
| 18142 | N030000 | Diabetic cheiroarthropathy |
| 42505 | C101z00 | Diabetes mellitus NOS with ketoacidosis |
| 39317 | C106100 | Diabetes mellitus, adult onset, + neurological manifestation |
| 47584 | F420500 | Advanced diabetic retinal disease |
| 32556 | C107.12 | Diabetes with gangrene |
| 48078 | F372000 | Acute painful diabetic neuropathy |
| 64357 | C10zz00 | Diabetes mellitus NOS with unspecified complication |
| 33343 | C10y.00 | Diabetes mellitus with other specified manifestation |
| 17313 | F440700 | Diabetic iritis |
| 45491 | C10z.00 | Diabetes mellitus with unspecified complication |
| 65025 | C107z00 | Diabetes mellitus NOS with peripheral circulatory disorder |
| 33807 | C107200 | Diabetes mellitus, adult with gangrene |
| 39420 | F381300 | Myasthenic syndrome due to diabetic amyotrophy |
| 52236 | C10A.00 | Malnutrition-related diabetes mellitus |
| 54856 | C101100 | Diabetes mellitus, adult onset, with ketoacidosis |
| 35105 | C104100 | Diabetes mellitus, adult onset, with renal manifestation |
| 62384 | 2G5V.00 | O/E - right chronic diabetic foot ulcer |
| 11848 | C314.11 | Renal diabetes |
| 43139 | C102100 | Diabetes mellitus, adult onset, with hyperosmolar coma |
| 43857 | C10M.00 | Lipoatrophic diabetes mellitus |
| 57333 | N030011 | Diabetic cheiropathy |
| 63357 | C107100 | Diabetes mellitus, adult, + peripheral circulatory disorder |
| 13279 | C104y00 | Other specified diabetes mellitus with renal complications |
| 34283 | C105z00 | Diabetes mellitus NOS with ophthalmic manifestation |
| 55431 | L180X00 | Pre-existing diabetes mellitus, unspecified |
| 61122 | C10H.00 | Diabetes mellitus induced by non-steroid drugs |
| 70821 | C10yz00 | Diabetes mellitus NOS with other specified manifestation |
| 64283 | C10zy00 | Other specified diabetes mellitus with unspecified comps |
| 43453 | C10C.00 | Diabetes mellitus autosomal dominant |
| 46290 | C108y00 | Other specified diabetes mellitus with multiple comps |
| 65062 | C103z00 | Diabetes mellitus NOS with ketoacidotic coma |
| 94383 | C10N000 | Secondary diabetes mellitus without complication |
| 16491 | C106.13 | Diabetes mellitus with polyneuropathy |
| 59903 | C106.11 | Diabetic amyotrophy |
| 41686 | Cyu2000 | [X]Other specified diabetes mellitus |
| 47377 | C105y00 | Other specified diabetes mellitus with ophthalmic complicatn |
| 59288 | C103y00 | Other specified diabetes mellitus with coma |
| 60046 | C135.12 | Diabetes insipidus - pituitary |
| 63371 | C10y100 | Diabetes mellitus, adult, + other specified manifestation |
| 66675 | C10A000 | Malnutrition-related diabetes mellitus with coma |
| 68843 | C103100 | Diabetes mellitus, adult onset, with ketoacidotic coma |
| 64449 | C108z00 | Unspecified diabetes mellitus with multiple complications |
| 95539 | C10FS00 | Maternally inherited diabetes mellitus |
| 96506 | C10G000 | Secondary pancreatic diabetes mellitus without complication |
| **Osteoporosis/ostopenia** | | |
| 28882 | 8HTS.00 | Referral to osteoporosis clinic |
| 10359 | 66a1.00 | Follow-up osteoporosis assessment |
| 38395 | N331B00 | Postmenopausal osteoporosis with pathological fracture |
| 16857 | N330C00 | Osteoporosis localized to spine |
| 33526 | N331300 | Osteoporosis of disuse with pathological fracture |
| 68019 | N331400 | Postsurgical malabsorption osteoporosis with path fracture |
| 37646 | 66a2.00 | Osteoporosis treatment started |
| 40428 | N330300 | Idiopathic osteoporosis |
| 36644 | 66a3.00 | Osteoporosis treatment stopped |
| 34798 | N330z00 | Osteoporosis NOS |
| 60433 | N330900 | Osteoporosis in multiple myelomatosis |
| 14967 | N330000 | Osteoporosis, unspecified |
| 93655 | N330700 | Postsurgical malabsorption osteoporosis |
| 18265 | 9N0h.00 | Seen in osteoporosis clinic |
| 18825 | NyuB800 | [X]Unspecified osteoporosis with pathological fracture |
| 45736 | N331H00 | Collapse of cervical vertebra due to osteoporosis |
| 12673 | N331900 | Osteoporosis + pathological fracture thoracic vertebrae |
| 48772 | N331A00 | Osteoporosis + pathological fracture cervical vertebrae |
| 25650 | N330D00 | Osteoporosis due to corticosteroids |
| 57301 | NyuB000 | [X]Other osteoporosis with pathological fracture |
| 105290 | 9Od6.00 | Osteoporosis monitoring verbal invitation |
| 41376 | 66a8.00 | Osteoporosis - exercise advice |
| 98433 | 9kj..00 | Osteoporosis - enhanced services administration |
| 16307 | N330100 | Senile osteoporosis |
| 68122 | 9Od3.00 | Osteoporosis monitoring first letter |
| 277 | N330.00 | Osteoporosis |
| 39596 | 66aE.00 | Refer to osteoporosis specialist |
| 93455 | 9Od4.00 | Osteoporosis monitoring second letter |
| 17377 | N331800 | Osteoporosis + pathological fracture lumbar vertebrae |
| 41755 | NyuB100 | [X]Other osteoporosis |
| 4013 | N331L00 | Collapse of vertebra due to osteoporosis NOS |
| 93705 | N331M11 | Minimal trauma fracture due to unspecified osteoporosis |
| 48962 | 66a5.00 | Osteoporosis - no treatment |
| 9700 | N330200 | Postmenopausal osteoporosis |
| 38903 | 66a7.00 | Osteoporosis - dietary assessment |
| 26292 | 66a9.00 | Osteoporosis - falls prevention |
| 98189 | 66aB.00 | Osteoporosis - no treatment response |
| 89922 | 8I6c.00 | Osteoporosis treatment not indicated |
| 98760 | 9kj0.00 | Bone sparing drug treatment offered for osteoporosis - ESA |
| 99817 | 14GB.00 | History of osteoporosis |
| 27597 | N331600 | Idiopathic osteoporosis with pathological fracture |
| 104186 | 9hP1.00 | Excepted osteoporosis quality indicators: informed dissent |
| 39334 | N331200 | Postoophorectomy osteoporosis with pathological fracture |
| 92887 | 9Od2.00 | Osteoporosis monitoring default |
| 11503 | N331M00 | Fragility fracture due to unspecified osteoporosis |
| 101443 | 9hP0.00 | Excepted osteoporosis quality indicators: patient unsuitable |
| 62702 | N330400 | Dissuse osteoporosis |
| 19048 | N331K00 | Collapse of thoracic vertebra due to osteoporosis |
| 25534 | 9Od9.00 | Osteoporosis monitoring check done |
| 31580 | N330A00 | Osteoporosis in endocrine disorders |
| 65163 | 9Od0.00 | Attends osteoporosis monitoring |
| 24093 | N330500 | Drug-induced osteoporosis |
| 34129 | 66a4.00 | Osteoporosis treatment changed |
| 5841 | N331J00 | Collapse of lumbar vertebra due to osteoporosis |
| 102730 | NyuB200 | [X]Osteoporosis in other disorders classified elsewhere |
| 102169 | 9hP..00 | Exception reporting: osteoporosis quality indicators |
| 46894 | N331500 | Drug-induced osteoporosis with pathological fracture |
| 70233 | 66aA.00 | Osteoporosis - treatment response |
| 36796 | 9Od..00 | Osteoporosis monitoring administration |
| 54232 | N330800 | Localized osteoporosis - Lequesne |
| 3346 | N330B00 | Vertebral osteoporosis |
| 70349 | N330600 | Postoophorectomy osteoporosis |
| 11603 | 66a..00 | Osteoporosis monitoring |
| 61121 | 9Od7.00 | Osteoporosis monitoring telephone invitation |
| 102017 | 9Od5.00 | Osteoporosis monitoring third letter |
| 7438 | NyuBC00 | [X]Osteopenia |
| **Eosinophilia^†^** | | |
| 67479 | D403200 | Drug induced eosinophilia |
| 5495 | D403.00 | Eosinophilia |
| 55214 | D403z00 | Eosinophilia NOS |
| 52907 | D403400 | Secondary eosinophilia NOS |
| 63510 | 4E32.00 | Sputum: eosinophilia |
| 16439 | H583100 | Tropical eosinophilia |
| 59062 | D403100 | Idiopathic eosinophilia |
| 22915 | H583.00 | Pulmonary eosinophilia |
| 53414 | D403300 | Allergic eosinophilia |
| 20269 | H583z00 | Pulmonary eosinophilia NOS |
| **Pneumonia** | | |
| 572 | H26..00 | Pneumonia due to unspecified organism |
| 886 | H25..00 | Bronchopneumonia due to unspecified organism |
| 9639 | H260.00 | Lobar pneumonia due to unspecified organism |
| 3683 | H261.00 | Basal pneumonia due to unspecified organism |
| 104121 | H2B..00 | Community acquired pneumonia |
| 4910 | H56y100 | Interstitial pneumonia |
| 103475 | H564.11 | Cryptogenic organising pneumonia |
| **Smoking** | | |
| 33 | 1371 | Never smoked tobacco |
| 11788 | 1371.11 | Non-smoker |
| 60 | 137L.00 | Current non-smoker |
| 52503 | 13WK.00 | No smokers in the household |
| 98177 | 9kn..00 | Non-smoker annual review - enhanced services administration |
| 12961 | 1377 | Ex-trivial smoker (<1/day) |
| 12957 | 1378 | Ex-light smoker (1-9/day) |
| 12955 | 1379 | Ex-moderate smoker (10-19/day) |
| 12956 | 137A.00 | Ex-heavy smoker (20-39/day) |
| 12959 | 137B.00 | Ex-very heavy smoker (40+/day) |
| 12946 | 137F.00 | Ex-smoker - amount unknown |
| 776 | 137K.00 | Stopped smoking |
| 26470 | 137N.00 | Ex pipe smoker |
| 19488 | 137O.00 | Ex cigar smoker |
| 90 | 137S.00 | Ex smoker |
| 12878 | 137T.00 | Date ceased smoking |
| 97210 | 137j.00 | Ex-cigarette smoker |
| 98447 | 9km..00 | Ex-smoker annual review - enhanced services administration |
| 72706 | E251300 | Tobacco dependence in remission |
| 16717 | H310100 | Smokers' cough |
| 72700 | ZV11600 | [V]Personal history of tobacco abuse |
| 12942 | 137..11 | Smoker - amount smoked |
| 12958 | 1372 | Trivial smoker - < 1 cig/day |
| 12941 | 1372.11 | Occasional smoker |
| 12944 | 1373 | Light smoker - 1-9 cigs/day |
| 1878 | 1374 | Moderate smoker - 10-19 cigs/d |
| 3568 | 1375 | Heavy smoker - 20-39 cigs/day |
| 1822 | 1376 | Very heavy smoker - 40+cigs/d |
| 12964 | 137C.00 | Keeps trying to stop smoking |
| 12240 | 137G.00 | Trying to give up smoking |
| 12947 | 137H.00 | Pipe smoker |
| 12943 | 137J.00 | Cigar smoker |
| 12945 | 137M.00 | Rolls own cigarettes |
| 93 | 137P.00 | Cigarette smoker |
| 1823 | 137P.11 | Smoker |
| 12952 | 137Q.00 | Smoking started |
| 12951 | 137Q.11 | Smoking restarted |
| 10558 | 137R.00 | Current smoker |
| 12966 | 137V.00 | Smoking reduced |
| 12965 | 137X.00 | Cigarette consumption |
| 12963 | 137Y.00 | Cigar consumption |
| 12960 | 137Z.00 | Tobacco consumption NOS |
| 12967 | 137a.00 | Pipe tobacco consumption |
| 31114 | 137b.00 | Ready to stop smoking |
| 30423 | 137c.00 | Thinking about stopping smoking |
| 30762 | 137d.00 | Not interested in stopping smoking |
| 41979 | 137e.00 | Smoking restarted |
| 46321 | 137f.00 | Reason for restarting smoking |
| 62686 | 137h.00 | Minutes from waking to first tobacco consumption |
| 10211 | 13p..00 | Smoking cessation milestones |
| 34126 | 13p0.00 | Negotiated date for cessation of smoking |
| 34127 | 13p1.00 | Smoking status at 4 weeks |
| 34374 | 13p2.00 | Smoking status between 4 and 52 weeks |
| 41405 | 13p3.00 | Smoking status at 52 weeks |
| 10898 | 13p4.00 | Smoking free weeks |
| 38112 | 13p5.00 | Smoking cessation programme start date |
| 28886 | 13p6.00 | Carbon monoxide reading at 4 weeks |
| 97643 | 38DH.00 | Fagerstrom test for nicotine dependence |
| 10184 | 67A3.00 | Pregnancy smoking advice |
| 98137 | 67H6.00 | Brief intervention for smoking cessation |
| 74907 | 745H.00 | Smoking cessation therapy |
| 81440 | 745H000 | Nicotine replacement therapy using nicotine patches |
| 85975 | 745H100 | Nicotine replacement therapy using nicotine gum |
| 85247 | 745H200 | Nicotine replacement therapy using nicotine inhalator |
| 89464 | 745H300 | Nicotine replacement therapy using nicotine lozenges |
| 94958 | 745H400 | Smoking cessation drug therapy |
| 91708 | 745Hy00 | Other specified smoking cessation therapy |
| 90522 | 745Hz00 | Smoking cessation therapy NOS |
| 9833 | 8B2B.00 | Nicotine replacement therapy |
| 32572 | 8B3Y.00 | Over the counter nicotine replacement therapy |
| 25106 | 8B3f.00 | Nicotine replacement therapy provided free |
| 67178 | 8BP3.00 | Nicotine replacement therapy provided by community pharmacis |
| 7622 | 8CAL.00 | Smoking cessation advice |
| 41042 | 8CAg.00 | Smoking cessation advice provided by community pharmacist |
| 18573 | 8H7i.00 | Referral to smoking cessation advisor |
| 98245 | 8HBM.00 | Stop smoking face to face follow-up |
| 10742 | 8HTK.00 | Referral to stop-smoking clinic |
| 98154 | 8HkQ.00 | Referral to NHS stop smoking service |
| 66409 | 8I2I.00 | Nicotine replacement therapy contraindicated |
| 63717 | 8I2J.00 | Bupropion contraindicated |
| 24529 | 8I39.00 | Nicotine replacement therapy refused |
| 57639 | 8I3M.00 | Bupropion refused |
| 11356 | 9N2k.00 | Seen by smoking cessation advisor |
| 11527 | 9N4M.00 | DNA - Did not attend smoking cessation clinic |
| 28834 | 9OO..00 | Anti-smoking monitoring admin. |
| 32083 | 9OO..11 | Stop smoking clinic admin. |
| 7130 | 9OO..12 | Stop smoking monitoring admin. |
| 12953 | 9OO1.00 | Attends stop smoking monitor. |
| 40418 | 9OO2.00 | Refuses stop smoking monitor |
| 40417 | 9OO3.00 | Stop smoking monitor default |
| 42722 | 9OO4.00 | Stop smoking monitor 1st lettr |
| 60720 | 9OO5.00 | Stop smoking monitor 2nd lettr |
| 66387 | 9OO6.00 | Stop smoking monitor 3rd lettr |
| 53101 | 9OO7.00 | Stop smoking monitor verb.inv. |
| 58597 | 9OO8.00 | Stop smoking monitor phone inv |
| 63901 | 9OO9.00 | Stop smoking monitoring delete |
| 19485 | 9OOA.00 | Stop smoking monitor.chck done |
| 21637 | 9OOZ.00 | Stop smoking monitor admin.NOS |
| 96992 | 9kc..00 | Smoking cessation - enhanced services administration |
| 98493 | 9kc0.00 | Smoking cessatn monitor template complet - enhanc serv admin |
| 98347 | 9ko..00 | Current smoker annual review - enhanced services admin |
| 6359 | E023.00 | Nicotine withdrawal |
| 32687 | E251.00 | Tobacco dependence |
| 95610 | E251000 | Tobacco dependence, unspecified |
| 70746 | E251100 | Tobacco dependence, continuous |
| 68658 | E251z00 | Tobacco dependence NOS |
| 61905 | Eu17.00 | [X]Mental and behavioural disorder due to use of tobacco |
| 56144 | Eu17100 | [X]Mental and behav dis due to use of tobacco: harmful use |
| 9045 | ZG23300 | Advice on smoking |
| 63666 | ZRBm200 | Fagerstrom test for nicotine dependence |
| 63299 | ZRBm211 | FTND - Fagerstrom test for nicotine dependence |
| 47273 | ZRaM.00 | Motives for smoking scale |
| 91513 | ZRao.00 | Occasions for smoking scale |
| 59866 | ZRh4.00 | Reasons for smoking scale |
| 49418 | ZRh4.11 | RFS - Reasons for smoking scale |
| 12954 | ZV4K000 | [V]Tobacco use |
| 35055 | ZV6D800 | [V]Tobacco abuse counselling |
| **FEV_1_** | | |
| 14453 | 3397 | Forced expiratory volume - FEV |
| 10320 | 339O.00 | Forced expired volume in 1 second |
| 23237 | 339a.00 | FEV1 before bronchodilation |
| 107044 | 3397200 | FEV1 after change of bronchodilator |
| 19830 | 339b.00 | FEV1 after bronchodilation |
| **Exacerbations** | | |
| 100123 | 8BP8.00 | Antibiotic therapy for acute pulmonary exac. |
| 19106 | 66Yd.00 | COPD accident and emergency |
| 19003 | 66Ye.00 | Emergency COPD admission since last appointment |
| 11019 | 8H2R.00 | Admit COPD emergency |
| 7884 | H3y1.00 | COPD with acute exacerbation |
| 1446 | H312200 | Acute exacerbation of COPD |
| 46036 | 66Yi.00 | Multiple COPD emergency hospital admissions |

FEV_1_: forced expiratory volume in 1 second

†Eosinophilia was defined using a Read code for diagnosis of eosinophilia in the medical history or a record of blood eosinophil count <0.4×10^9^ cells/L using the record closest to COPD diagnosis and no more than three months after.

Table S6: Comorbidity test variables

| **Comorbidity test** | **Enttype variable** |
| --- | --- |
| FEV_1_^†^ | Enttype 394 |
| FEV_1_/FVC | Enttype 395 |
| FVC | Enttype 396 |
| Eosinophil count | Enttype 168 |

†Note that for patients with insufficient record of FEV_1_ % predicted, this was calculated using the ECCS equation published by Roca et al, based on FEV_1_ test files.^(^[^2^](#_ENREF_2)^)^

Figure S4: Distribution of year of diagnosis by region


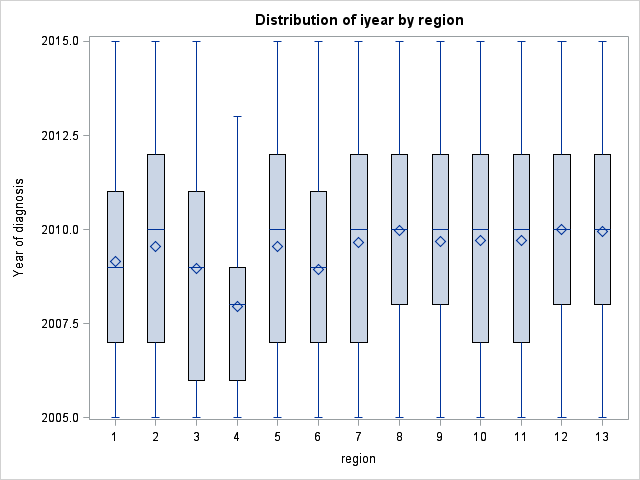


1 = North East; 2 = North West; 3 = Yorkshire & The Humber; 4 = East Midlands; 5 = West Midlands; 6 = East of England; 7 = South West; 8 = South Central; 9 = London; 10 = South East Coast; 11 = Northern Ireland; 12 = Scotland; 13 = Wales

**References**

1. Kuster SP, Kuster D, Schindler C, Rochat MK, Braun J, Held L, et al. Reference equations for lung function screening of healthy never-smoking adults aged 18–80 years. European Respiratory Journal. 2008;31(4):860-8.

2. Roca J, Burgos F, Sunyer J, Saez M, Chinn S, Anto JM, et al. References values for forced spirometry. Group of the European Community Respiratory Health Survey. The European respiratory journal. 1998;11(6):1354-62.
